# Exploration of hydroxymethylation in Kagami-Ogata syndrome caused by hypermethylation of imprinting control regions

**DOI:** 10.1186/s13148-015-0124-y

**Published:** 2015-08-28

**Authors:** Keiko Matsubara, Masayo Kagami, Kazuhiko Nakabayashi, Kenichiro Hata, Maki Fukami, Tsutomu Ogata, Kazuki Yamazawa

**Affiliations:** Department of Molecular Endocrinology, National Research Institute for Child Health and Development, 2-10-1 Okura, Setagaya, Tokyo 157-8535 Japan; Department of Maternal-Fetal Biology, National Research Institute for Child Health and Development, 2-10-1 Okura, Setagaya, Tokyo 157-8535 Japan; Department of Pediatrics, Hamamatsu University School of Medicine, 1-20-1 Handayama, Higashi, Hamamatsu, Shizuoka 431-3192 Japan; Department of Pediatrics, Keio University School of Medicine, 35 Shinanomachi, Shinjuku, Tokyo 160-8582 Japan; Clinical Genetics Center, National Hospital Organization Tokyo Medical Center, 2-5-1 Higashigaoka, Meguro, Tokyo 152-8902 Japan

**Keywords:** Methylation, Hydroxymethylation, Oxidative bisulfite, Kagami-Ogata syndrome, Imprinting, Methylation array

## Abstract

**Background:**

5-Hydroxymethylcytosine (5hmC), converted from 5-methylcytosine (5mC) by ten-eleven translocation (Tet) enzymes, has recently drawn attention as the “sixth base” of DNA since it is considered an intermediate of the demethylation pathway. Nonetheless, it remains to be addressed how 5hmC is linked to the development of human imprinting disorders. In this regard, conventional bisulfite (BS) treatment is unable to differentiate 5hmC from 5mC. It is thus hypothesized that BS conversion-derived “hypermethylation” at imprinting control regions (ICRs), which may cause imprinting disorders, would in fact be attributable to excessively increased levels of 5hmC as well as 5mC. To test this hypothesis, we applied the newly developed oxidative BS (oxBS) treatment to detect 5hmC in blood samples from Kagami-Ogata syndrome (KOS14) patients caused by an epimutation (hypermethylation) of two differentially methylated regions (DMRs) functioning as ICRs, namely, IG-DMR and *MEG3*-DMR.

**Findings:**

oxBS with pyrosequencing revealed that there were few amounts of 5hmC at the hypermethylated IG-DMR and *MEG3*-DMR in blood samples from KOS14 patients. oxBS with genome-wide methylation array demonstrated that global levels of 5hmC were very low with similar distribution patterns in blood samples from KOS14 patients and normal controls. We also confirmed the presence of large amounts of 5hmC in the brain sample from a normal control.

**Conclusions:**

5hmC is not a major component in abnormally hypermethylated ICRs or at a global level, at least in blood from KOS14 patients. As the brain sample contained large amounts of 5hmC, the neural tissues of KOS14 patients are promising candidates for analysis in elucidating the role of 5hmC in the neurodevelopmental context.

**Electronic supplementary material:**

The online version of this article (doi:10.1186/s13148-015-0124-y) contains supplementary material, which is available to authorized users.

## Findings

### Background

DNA methylation is a major epigenetic modification of the mammalian genome that plays a vital role in cellular processes including gene expression, retrotransposon silencing, X chromosome inactivation, and genomic imprinting [1, 2]. Recent studies have highlighted the ability of ten-eleven translocation (Tet) family proteins to enzymatically convert 5-methylcytosine (5mC) to 5-hydroxymethylcytosine (5hmC) through Fe(II)/α-ketoglutarate-dependent hydroxylation [3–5]. Additional oxidative steps to generate 5-formylcytosine (5fC) and 5-carboxylcytosine (5caC), and the following base excision repair mechanism would lead to removal of the methylated base and its replacement with an unmethylated cytosine (C) in a DNA replication-independent manner [5–9]. Alternatively, 5hmC converted from 5mC by Tet enzymes can be diluted through subsequent rounds of DNA replication and cell division due to the low enzymatic activity of Dnmt1 on the hemi-hydroxymethylated DNA [10]. Tet-driven 5mC conversion to 5hmC thus provides a direct mechanistic pathway for both active and passive DNA demethylation, suggesting an important role of 5hmC in diverse biological processes through the regulation of DNA methylation states.

Methylation levels of cytosines have usually been evaluated using a reaction with sodium bisulfite (BS) followed by PCR amplification [11]. BS treatment yields deamination of C to uracil that is read as thymine (T) in subsequent assays. In contrast, 5mC is resistant to deamination by BS and is thus read as C. In this regard, it is noteworthy that this BS treatment does not distinguish between 5mC and 5hmC because 5hmC is also resistant to deamination [12, 13], raising the possibility that BS-based analyses so far could potentially overestimate 5mC levels in a given sample due to the presence of 5hmC.

Thus far, our laboratory has extensively investigated the features of human imprinting disorders mainly attributable to abnormal methylation, especially Kagami-Ogata syndrome (KOS14), which is characterized by a unique array of clinical features including facial abnormalities, a small bell-shaped thorax with coat-hanger appearance of the ribs, abdominal wall defects, placentomegaly, and polyhydramnios [14–17]. One of the causative molecular mechanisms of KOS14 is aberrant hypermethylation of the paternally methylated differentially methylated regions (DMRs) functioning as imprinting control regions (ICRs), i.e., the germline-derived IG-DMR and postfertilization-derived *MEG3*-DMR, which perturbs the expression of clustered imprinted genes on 14q32.2. In particular, the markedly elevated *RTL1* expression primarily causes the KOS14 phenotype [14, 15]. However, to date, there have been no reports that investigate the functional role of hydroxymethylation in human imprinting disorders caused by abnormal hypermethylation.

To clarify this issue, we utilized the newly developed modified bisulfite technique in conjunction with the high-throughput Infinium HumanMethylation450 BeadChip, as well as pyrosequencing and cloning-based sequencing, to detect the 5hmC level in KOS14 blood samples caused by imprinting defects due to hypermethylation of the IG-DMR and *MEG3*-DMR. In particular, we tested the hypothesis that conventional BS conversion-derived “hypermethylation” could in fact be attributable to excessively increased levels of 5hmC as well as 5mC. In this context, based on the notion that 5hmC functions as an intermediate in an active DNA demethylation pathway by Tet-driven oxidative steps, 5hmC could be generated at the ICRs, perhaps as a consequence of a self-correcting mechanism that allows abnormally hypermethylated alleles to be demethylated.

### Methods summary

Detailed methods are available in Additional file 1.

#### Samples

We analyzed blood genomic DNA from three patients molecularly diagnosed as KOS14 with an epimutation (hypermethylation) involving the IG-DMR and *MEG3*-DMR, pooled blood genomic DNA from ten normal control subjects, and purchased human brain genomic DNA. All procedures followed were in accordance with the ethical approval granted by the institutional review boards at the National Center for Child Health and Development (project 518), and written informed consent was obtained from all participants.

#### BS/oxidative BS conversion

We applied oxidative BS (oxBS) treatment whereby selective chemical oxidation converts 5hmC into 5fC, and subsequent BS treatment converts 5fC and C into uracil (later T). Consequently, the “methylation” level derived by the BS treatment is the combined levels of 5mC and 5hmC, while the oxBS treatment gives the level of 5mC alone. The 5hmC value is thus derived by subtracting the oxBS level from the BS level [18].

#### oxBS pyrosequencing, cloning-based sequencing, and methylation array analysis

First, oxBS pyrosequencing was carried out to analyze methylation and hydroxymethylation status at the IG-DMR and *MEG3*-DMR with BS- and oxBS-treated samples. Because the 450K BeadChip probes described below did not cover the IG-DMR region, cloning-based sequencing for the IG-DMR was also performed.

Next, the BS- or oxBS-treated samples were applied to an Infinium HumanMethylation450 BeadChip Kit (Illumina) and processed following the manufacturer’s recommendations (BS/oxBS-array). The methylation level at each probe was represented by *β* values ranging from 0 (completely unmethylated) to 1 (completely methylated). 5hmC levels were thus calculated by subtraction (Δ*β*) of the *β* value of oxBS-treated samples, representing 5mC only, from the *β* value of BS-treated samples, representing 5mC and 5hmC, at each probe site.

### Results

#### Methylation/hydroxymethylation at the IG-DMR and *MEG3*-DMR

**Fig. 1:**
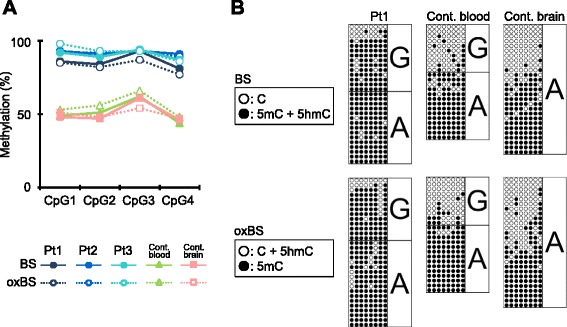
Methylation/hydroxymethylation analysis by BS and oxBS treatment at the IG-DMR. **a** Methylation values (%methylation) determined by pyrosequencing with BS or oxBS conversion for three KOS14 blood samples (Pt1–Pt3), one control pooled blood sample, and one control adult brain sample. *Solid lines* denote values with BS, whereas *dotted lines* denote those with oxBS. **b** Methylation profiles determined by cloning-based sequencing with BS or oxBS conversion for one KOS14 blood sample (Pt1), one control blood, and one control adult brain sample. For BS, *filled* and *open* circles indicate (methylated + hydroxymethylated) and unmethylated cytosines at the CpG sites, respectively, while for oxBS, *filled* and *open* circles indicate methylated and (hydroxymethylated + unmethylated) cytosines at the CpG sites, respectively. The typing data of A/G SNP (rs10133627) are also denoted. Note this control blood sample was derived from a single person, rather than a pooled blood sample

The pyrosequencing results revealed that, as expected, the “methylation” values derived by BS reaction at the IG-DMR and *MEG3*-DMR in KOS14 patients were considerably high compared to those in the blood/brain samples of the normal control (Fig. 1a and Additional file 2: Figure S1). The values derived by oxBS reaction at the IG-DMR and *MEG3*-DMR were also increased in KOS14 patients, whereas those in the control blood/brain were normal. The result of subtraction of the oxBS values from the BS values, which corresponds to the 5hmC level at each CpG, was nearly zero in all KOS14 patients, as well as the control blood/brain samples.

The results of BS/oxBS cloning-based sequencing for the IG-DMR were consistent with those of pyrosequencing, i.e., there were no apparent differences between the dot patterns of BS and oxBS treatment in blood samples from KOS14 and the normal control, indicating that 5hmC rarely existed at the IG-DMR in blood samples (Fig. 1b). Of note, some CpG sites in the brain sample from the control showed disparity in methylation levels between BS and oxBS treatment, suggesting that some 5hmC distributed in a CpG site-specific manner only in the brain, though statistical comparisons were generally inapplicable in this cloning-based assay. It is also noteworthy that heterozygous SNP (rs10133627) allowed the discrimination of the parental origin of each clone in blood samples obtained from patient 1 and the normal control; however, parental origin-specific distribution of 5hmC was not observed in these samples.

#### BS/oxBS-array

Using a detection *P* value threshold of 0.01 to filter probes, we found that the technical replicates for both BS and oxBS conversion showed a strong correlation of *r* = 0.97–0.99 (Additional file 3: Figure S2), indicating a high degree of reproducibility. Thus, we used the average of these replicates as the final measurement of 5mC and 5hmC. Hierarchical clustering of arrays on *β* value showed a clear separation between blood and brain samples (Additional file 4: Figure S3). Note there were distinct differences between clusterings of BS and oxBS conversion samples in brain tissue, implying a considerable amount of 5hmC in the brain.

**Fig. 2:**
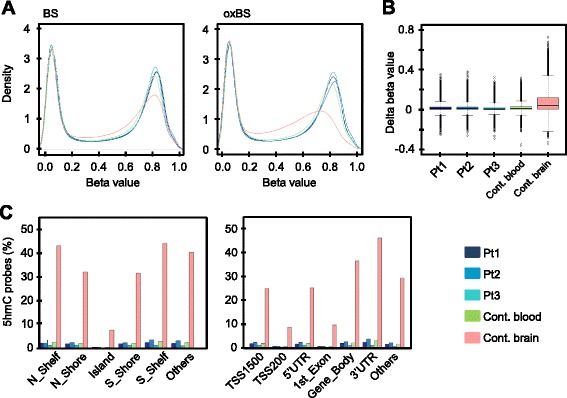
Representative data of BS/oxBS-array. **a** Density plots of normalized beta values with BS and oxBS conversion for three KOS14 blood samples (Pt1–Pt3), one control pooled blood sample, and one control adult brain sample. Beta values show a bimodal distribution for both BS and oxBS conversion samples. Of note, a left-skewed methylated peak is observed in the oxBS brain sample. **b** Box and whisker plots of Δ*β* score (corresponding to 5hmC value) for each sample. **c** Distribution of probes with Δ*β* ≥ 0.1 according to genomic features for each sample

Density plots of normalized *β* values for three KOS14 blood samples, one control pooled blood sample, and one adult brain sample represented a bimodal distribution for both BS and oxBS conversion samples (Fig. 2a). It is also noteworthy that the methylated peaks (larger *β* value peaks) were left-skewed only in the oxBS brain sample, corresponding to lower global methylation/higher hydroxymethylation levels in the brain. No difference between the three KOS14 blood samples and the control pooled blood sample was observed.

Distribution of the Δ*β* score (as a reflection of the 5hmC level at each particular probe position) of each sample is shown in Fig. 2b. The median level of Δ*β* in the brain sample was higher than those in blood samples from KOS14 patients and pooled controls, which were very close to zero. Table 1 shows the number of probes with Δ*β* ≥ 0.1. While 28.3 % of the probes showed Δ*β* ≥ 0.1 in the control brain sample, only 1–3 % of the probes with Δ*β* ≥ 0.1 were observed in blood samples from KOS14 patients, as well as the normal controls. The number of probes with Δ*β* ≥ 0.1 was similar in blood samples from KOS14 patients and from the normal controls. Notably, we investigated six specific individual CpG sites where high levels of 5hmC were previously validated in brain samples by Stewart et al. [19], and confirmed substantial amounts of 5hmC at the same sites only in the brain sample in our assay as well (Additional file 5: Table S1).

**Table 1:** Number of probes with Δ*β* ≥ 0.1

	Δ*β* ≥ 0.3	0.3 > Δ*β* ≥ 0.2	0.2 > Δ*β* ≥ 0.1	Total number of probes with Δ*β* ≥ 0.1
Pt1 (blood)	7	197	6309	6513 (1.5 %)
Pt2 (blood)	8	296	8819	9123 (2.1 %)
Pt3 (blood)	3	68	3416	3487 (0.81 %)
Control (blood)	2	147	7026	7175 (1.7 %)
Control (brain)	8215	30,652	83,294	122,161 (28.3 %)

We next investigated the distribution of probes with Δ*β* ≥ 0.1, i.e., hydroxymethylated probes according to genomic features (Fig. 2c). Although the overall proportion of hydroxymethylated probes was much higher in the brain sample than in the blood samples, distribution patterns of hydroxymethylated probes in genomic regions were similar among all samples analyzed. Overall, the proportion of hydroxymethylated probes showed a decrease at the CpG islands, and at TSS 200 and first exon regions in all samples, consistent with previous reports that demonstrated an inverse correlation between 5hmC and CpG density at the promoter [18–20].

Afterwards, we looked into the average *β* values of 32 probes encompassing the *MEG3*-DMR (Additional file 6: Figure S4A). The average *β* values of both BS- and oxBS-treated samples were higher in patients with KOS14 than in the normal control blood/brain. However, Δ*β* values were remarkably subtle, indicating that only small amounts of 5hmC existed at the *MEG3*-DMR in blood samples from three patients with KOS14 as well as the control pool, similarly at the IG-DMR and other genomic loci.

Finally, we inquired into the data of other DMRs outside chromosome 14 (Additional file 6: Figure S4B–H). Overall, there was no difference between the average *β* values of BS-treated blood samples from patients with KOS14 and those from the normal control, indicating that multiple methylation defects were not observed in these three patients. Moreover, Δ*β* values were remarkably subtle in all blood samples analyzed, demonstrating that small amounts of 5hmC existed at other DMRs as well as at the IG-DMR and *MEG3*-DMR in blood. Of note, numerous probes had relatively high Δ*β* values, especially at the *PEG10*-DMR, *MEST*-DMR, and *GNAS* exon A/B-DMR, suggesting that substantial amounts of 5hmC were included at these DMRs in the brain.

### Discussion

In this study, we analyzed the 5hmC level and distribution pattern using the newly developed oxBS treatment combined with pyrosequencing, cloning-based sequencing, and the 450K methylation array in peripheral blood taken from three patients with KOS14 and normal controls, and in the brain sample from the normal control. This is the first study to investigate hydroxymethylation in human imprinting disorders caused by abnormal hypermethylation at ICRs and provides several notable findings.

First, our study found few amounts of 5hmC at the IG-DMR and *MEG3*-DMR as well as at other DMRs or a genome-wide level in blood samples from KOS14 patients. Although we hypothesized that conventional BS conversion-derived “hypermethylation” would be in fact attributable to excessively increased levels of 5hmC in addition to 5mC, our data did not support this hypothesis. Taking into account that 5hmC has been proposed to be an intermediate in an active DNA demethylation pathway by Tet-mediated oxidative steps, 5hmC was assumed to be yielded perhaps as a consequence of a self-correcting mechanism, which enables abnormally hypermethylated alleles to be demethylated. However, this is not applicable at least to the blood samples, which contain a very small amount of 5hmC.

Second, BS/oxBS-array methods are powerful tools for (hydroxy)methylation profiling, suitable for high-throughput sample processing and genome-wide 5mC/5hmC mapping. In this context, Stewart et al. developed and utilized the oxBS-array to measure 5hmC in normal human blood and brain samples [19]. They validated that six individual CpG sites contained a substantial amount of hydroxymethylation in the brain. We confirmed abundant levels of 5hmC at the same sites only in the brain, suggesting that our assay worked properly. This is supported by the findings of a high degree of reproducibility in technical replicates and a clear separation between blood and brain samples in hierarchical clustering on *β* values.

Third, 5hmC in neural samples derived from KOS14 patients is a promising candidate for future analysis. In this regard, the highest levels of 5hmC are observed in neural tissues [13, 21]. Our BS/oxBS-array data have supported this at some DMRs, as well as at a genome-wide level. In addition, neurodevelopmental problems are one of the major symptoms of KOS14 [17]. Furthermore, several studies have recently shown that the dysregulation of 5hmC could be involved in neurological disorders such as Rett syndrome, fragile X syndrome, Huntington’s disease, and Alzheimer’s disease [22–25]. Although it seems to be difficult to obtain neural samples from KOS14 patients, induced pluripotent stem (iPS) cell-based disease modeling derived from KOS14 patients and its neural differentiation can be one of the most promising approaches.

In summary, this study is the first to demonstrate that, at least in the blood, 5hmC is not a major component of the abnormally hypermethylated ICR that causes human imprinting disorders. In addition, genome-wide analysis revealed that global levels of 5hmC were very subtle with similar distribution patterns in blood samples taken from KOS14 patients and normal controls. Given that a large amount of 5hmC exists in the brain, further studies of neural tissues are required to better understand the biological role of 5hmC in the neurodevelopmental context.

## Availability of supporting data

The data from this study have been submitted to the NCBI Gene Expression Omnibus (GEO; http://www.ncbi.nlm.nih.gov/geo/) under accession number GSE71328.

## Additional files

Additional file 1:
**Supplementary methods.** Further details on the methods used in this study (Additional file 7: Figure S5 and Additional file 8: Table S2). (DOCX 25.0 kb) Additional file 2: Figure S1. Methylation/hydroxymethylation analysis by BS and oxBS treatment at the *MEG3*-DMR by pyrosequencing. (PPB 63.5 kb) Additional file 3: Figure S2. Scatterplots showing correlation between technical replicates of beta values by BS/oxBS-array. (PPB 205 kb) Additional file 4: Figure S3. Hierarchical clustering analysis for BS/oxBS-array. (PPB 110 kb) Additional file 5: Table S1. Six probes with high Δβ values only in brain tissue. (XLSX 45.1 kb) Additional file 6: Figure S4. Methylation/hydroxymethylation analysis at the *MEG3*-DMR, *PLAGL1*-DMR, *PEG10*-DMR, *MEST*-DMR, *H19*-DMR, KvDMR1, *SNRPN*-DMR, and *GNAS* exon A/B-DMR by BS/oxBS-array. (PPB 240 kb) Additional file 7: Figure S5. Gel electropherograms showing appropriate digestion pattern of spike-in controls. (PPB 3.31 mb) Additional file 8: Table S2. Primer sequences utilized in BS/oxBS pyrosequencing and cloning-based sequencing. (XLSX 9.68 kb)

## Abbreviations

5caC: 5-carboxylcytosine
5fC: 5-formylcytosine
5hmC: 5-hydroxymethylcytosine
5mC: 5-methylcytosine
BS: sodium bisulfite
C: cytosine
DMR: differentially methylated region
ICR: imprinting control region
iPS cell: induced pluripotent stem cell
KOS14: Kagami-Ogata syndrome
oxBS: oxidative sodium bisulfite
Tet: ten-eleven translocation
