# Supplementary material for: Exploration of hydroxymethylation in Kagami-Ogata syndrome caused by hypermethylation of imprinting control regions
Source: Clin Epigenetics. 2015 Aug 28;7(1):90. doi: 10.1186/s13148-015-0124-y (PMC4552283; doi:10.1186/s13148-015-0124-y)
Supplement: Additional file 1: — Supplementary methods. Further details on the methods used in this study (Additional file 7: Figure S5 and Additional file 8: Table S2). (DOCX 25.0 kb) [file 13148_2015_124_MOESM1_ESM.docx]

**Supplementary methods**

**KOS14 and control samples**

We isolated genomic DNA from peripheral blood samples of three patients molecularly diagnosed as KOS14 with an epimutation (hypermethylation) affecting the normally unmethylated IG-DMR and *MEG3*-DMR of maternal origin (Patient 1, 12-month-old male; Patient 2, 1-month-old male; and Patient 3, 1-month-old female: Patient 1 was previously described [1] while Patients 2 and 3 were not reported). For comparison, pooled blood DNA from 10 normal control subjects (5 males and 5 females, ranging from 1 month to 6 years old) was used. Also, purchased human brain genomic DNA from a 41-year-old male (BioChain, catalogue #D1234035, lot #B801123) was analysed because brain tissue carries the highest level of 5hmC among all tissues analysed so far [2, 3], and thus the distribution of 5hmC in brain was of particular interest.

**BS/oxidative BS conversion**

To accurately detect and quantitate the levels of 5mC and 5hmC, we applied oxidative BS (oxBS) treatment according to the ‘High-input oxBS-450K protocol’ as previously described [4]. Briefly, 4 μg DNA in a total volume of 50 µl was sheared with a Bioruptor Pico (Diagenode) for 5 cycles of 30 sec on/30 sec off. 10 μl of sheared DNA was run on a 2% agarose gel to confirm DNA fragmentation (≤ 10 kb). The remaining fragmented DNA (40 μl) was split into 2 × 20 μl aliquots and purified using Agencourt AMPure XP (Beckman Coulter). Each sample was processed either through the oxBS or BS-only workflow as outlined in the TrueMethyl24® Kit User Guide (version 3.1, Cambridge Epigenetix). The oxBS sample was buffer-exchanged by P6 Micro Bio-Spin Columns (BioRad) and then subjected to oxidation, whereas the BS-only sample was subjected to mock oxidation. Subsequently, both samples were treated with sodium bisulfite reagents and finally eluted in 20 ul buffer. These procedures were repeated twice as technical replicates. Thus, in total, 4 samples (BS-1, -2, oxBS-1, and -2) were obtained from each specimen (three of blood DNA from patients with KOS14, one of blood DNA from the normal control pool, and one of human adult brain DNA) for further analysis.

To confirm that BS and oxBS conversion worked properly, we used the ‘Digestion Control’ as a spike-in control included in the kit. The Digestion Control contains a TaqI restriction site (5’TCGA3’), and the cytosine within this motif is 5hmC. Complete conversion of this base to uracil during the oxBS process will render the motif resistant to TaqI digestion, while the motif will digest following incomplete conversion or an oxBS failure. In contrast, complete BS conversion never changes the sequence of the motif, resulting in full digestion by TaqI, while incomplete or failed BS conversion will yield an undigested fragment. Thus, qualitative analysis by gel electrophoresis demonstrates the extent of BS/oxBS conversion. We had confirmed that all samples were properly converted through BS/oxBS treatment before proceeding to downstream assays (Additional file 7: Figure S5).

**oxBS pyrosequencing and cloning-based sequencing**

For pyrosequencing for the IG-DMR and *MEG3*-DMR, BS- or oxBS-treated samples were PCR-amplified using TaKaRa Ex Taq Hot Start Version (TaKaRa) with an annealing temperature of 54°C for 45 cycles. PCR products were subjected to pyrosequencing using a PyroMark Q24 pyrosequencing system (Qiagen) with the PyroMark Gold Q24 reagent kit. PyroMark Q24 software was used for data analysis. This assay was repeated twice as technical replicates.

For cloning-based sequencing for the IG-DMR, BS- or oxBS-treated samples were PCR-amplified using KAPA HiFi HotStart Uracil+ ReadyMix (KAPA Biosystems) with an annealing temperature of 57°C for 35 cycles. Subsequently, PCR products were A-tailed, subcloned with the TOPO TA Cloning Kit (Invitrogen), and then subjected to direct sequencing on the CEQ 8000 autosequencer (Beckman Coulter). The A/G SNP included in this region was genotyped simultaneously. Primer sequences for both assays are listed in Additional file 8: Table S2.

**450K BeadChip processing and data analysis**

7 μl of the BS- or oxBS-treated samples was applied to an Infinium HumanMethylation450 BeadChip kit (Illumina). Although these samples were spread across two arrays, 4 samples (BS-1, -2, oxBS-1, and -2) derived from one specimen were input into the same array to reduce inter-array differences between samples. We applied signal background subtraction, and inter-array variation was normalized using default control probes in GenomeStudio software (Illumina). Probes that fulfilled one or more of the following criteria were excluded: 1) with a detection P-value of > 0.01, 2) with no signal in one or more samples analysed, and 3) containing SNPs present in > 1% of the population (dbSNP137). We also excluded probes in sex chromosomes. After these filterings, 431,581 probes remained and were used in subsequent analyses.

The R package (version 3.0.2) was used for statistical analyses. Correlations between replicates were analysed by Spearman's rank test. The R package ‘*pvclust*’ was used for hierarchical clustering analysis.

**References**

1. Kagami M, Sekita Y, Nishimura G, Irie M, Kato F, Okada M et al. Deletions and epimutations affecting the human 14q32.2 imprinted region in individuals with paternal and maternal upd(14)-like phenotypes. Nat Genet. 2008;40:237-42.
2. Jin SG, Kadam S, Pfeifer GP. Examination of the specificity of DNA methylation profiling techniques towards 5-methylcytosine and 5-hydroxymethylcytosine. Nucleic Acids Res. 2010;38:e125.
3. Munzel M, Globisch D, Bruckl T, Wagner M, Welzmiller V, Michalakis S et al. Quantification of the sixth DNA base hydroxymethylcytosine in the brain. Angew Chem Int Ed Engl. 2010;49:5375-7.
4. Stewart SK, Morris TJ, Guilhamon P, Bulstrode H, Bachman M, Balasubramanian S et al. oxBS-450K: A method for analysing hydroxymethylation using 450K BeadChips. Methods. 2014;72:9-15.
