# Supplementary material for: Exploration of hydroxymethylation in Kagami-Ogata syndrome caused by hypermethylation of imprinting control regions
Source: Clin Epigenetics. 2015 Aug 28;7(1):90. doi: 10.1186/s13148-015-0124-y (PMC4552283; doi:10.1186/s13148-015-0124-y)
Supplement: Additional file 2: Figure S1. — Methylation/hydroxymethylation analysis by BS and oxBS treatment at the MEG3-DMR by pyrosequencing. (PPB 63.5 kb) [file 13148_2015_124_MOESM2_ESM.pptx]

## Slide 1
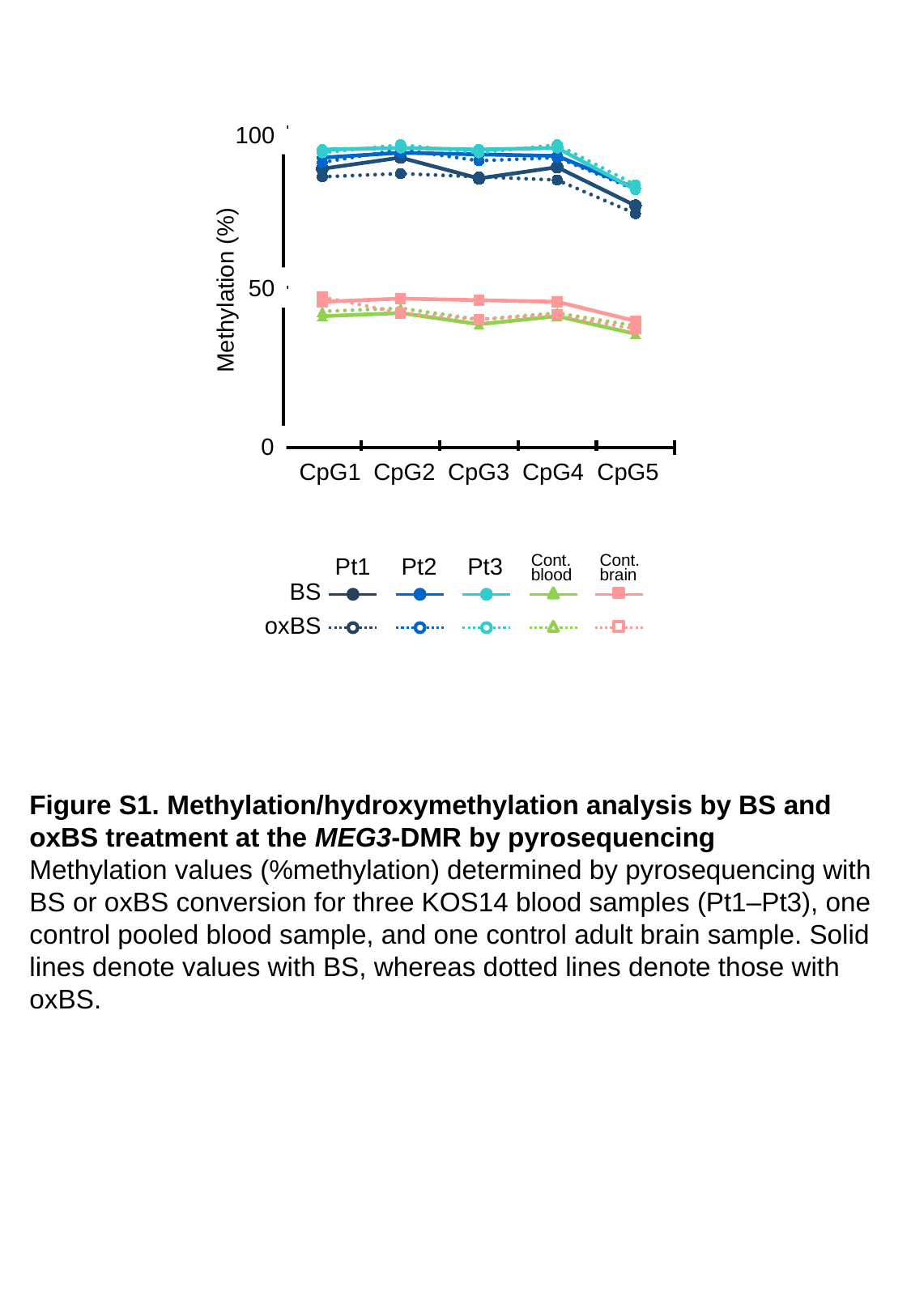

### Chart
| Category | Pt1 BS | Pt1 OX | Pt2 BS | Pt2 OX | Pt3 BS | Pt3 OX | Cont BS | Cont OX | Brain BS | Brain OX |
|---|---|---|---|---|---|---|---|---|---|---|
| P1 | 87.0 | 84.5 | 90.5 | 89.0 | 93.0 | 92.0 | 41.0 | 42.5 | 45.5 | 47.0 |
| P2 | 90.5 | 85.5 | 92.0 | 93.0 | 93.5 | 94.5 | 42.0 | 43.5 | 46.5 | 42.0 |
| P3 | 84.0 | 84.5 | 91.5 | 89.5 | 93.0 | 92.0 | 38.5 | 40.0 | 46.0 | 40.0 |
| P4 | 87.5 | 83.5 | 91.0 | 90.5 | 93.5 | 94.5 | 41.0 | 42.0 | 45.5 | 41.5 |
| P5 | 75.5 | 73.0 | 81.0 | 80.5 | 80.5 | 82.0 | 35.5 | 38.0 | 39.5 | 37.0 |100
50
Methylation (%)
0
CpG1
CpG2
CpG3
CpG4
CpG5
Pt1
Pt2
Pt3
Cont.
blood
Cont.
brain
BS
oxBS
Figure S1. Methylation/hydroxymethylation analysis by BS and oxBS treatment at the MEG3-DMR by pyrosequencing
Methylation values (%methylation) determined by pyrosequencing with BS or oxBS conversion for three KOS14 blood samples (Pt1–Pt3), one control pooled blood sample, and one control adult brain sample. Solid lines denote values with BS, whereas dotted lines denote those with oxBS.
