# Supplementary material for: Exploration of hydroxymethylation in Kagami-Ogata syndrome caused by hypermethylation of imprinting control regions
Source: Clin Epigenetics. 2015 Aug 28;7(1):90. doi: 10.1186/s13148-015-0124-y (PMC4552283; doi:10.1186/s13148-015-0124-y)
Supplement: Additional file 3: Figure S2. — Scatterplots showing correlation between technical replicates of beta values by BS/oxBS-array. (PPB 205 kb) [file 13148_2015_124_MOESM3_ESM.pptx]

## Slide 1
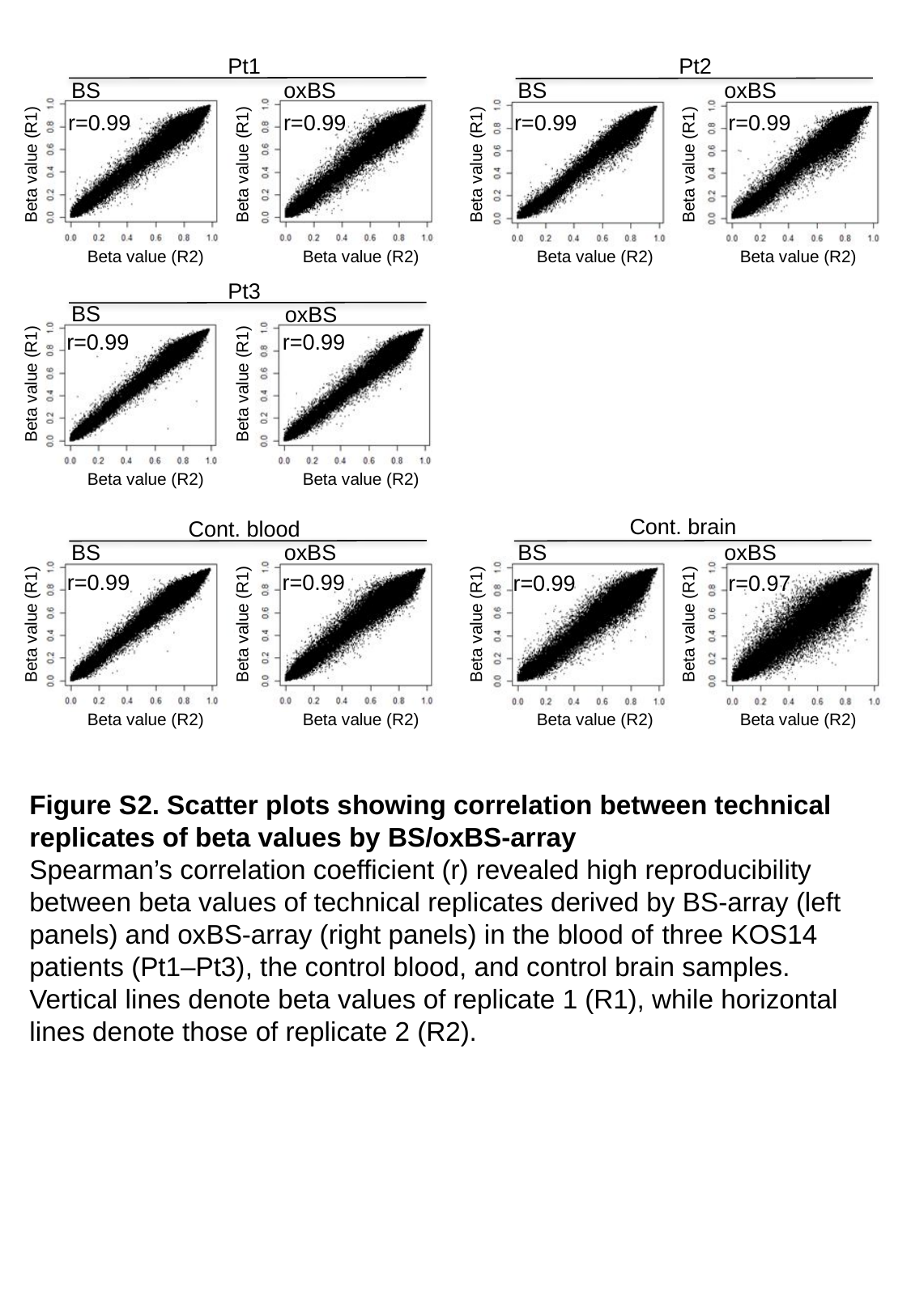

Pt1
Pt2
BS
oxBS
BS
oxBS
r=0.99
r=0.99
r=0.99
r=0.99
Beta value (R1)
Beta value (R1)
Beta value (R1)
Beta value (R1)
Beta value (R2)
Beta value (R2)
Beta value (R2)
Beta value (R2)
Pt3
BS
oxBS
r=0.99
r=0.99
Beta value (R1)
Beta value (R1)
Beta value (R2)
Beta value (R2)
Cont. brain
Cont. blood
r=0.99
r=0.99
r=0.99
r=0.97
BS
oxBS
BS
oxBS
Beta value (R1)
Beta value (R1)
Beta value (R1)
Beta value (R1)
Beta value (R2)
Beta value (R2)
Beta value (R2)
Beta value (R2)
Figure S2. Scatter plots showing correlation between technical replicates of beta values by BS/oxBS-array
Spearman’s correlation coefficient (r) revealed high reproducibility between beta values of technical replicates derived by BS-array (left panels) and oxBS-array (right panels) in the blood of three KOS14 patients (Pt1–Pt3), the control blood, and control brain samples. Vertical lines denote beta values of replicate 1 (R1), while horizontal lines denote those of replicate 2 (R2).
