# Supplementary material for: Exploration of hydroxymethylation in Kagami-Ogata syndrome caused by hypermethylation of imprinting control regions
Source: Clin Epigenetics. 2015 Aug 28;7(1):90. doi: 10.1186/s13148-015-0124-y (PMC4552283; doi:10.1186/s13148-015-0124-y)
Supplement: Additional file 4: Figure S3. — Hierarchical clustering analysis for BS/oxBS-array. (PPB 110 kb) [file 13148_2015_124_MOESM4_ESM.pptx]

## Slide 1
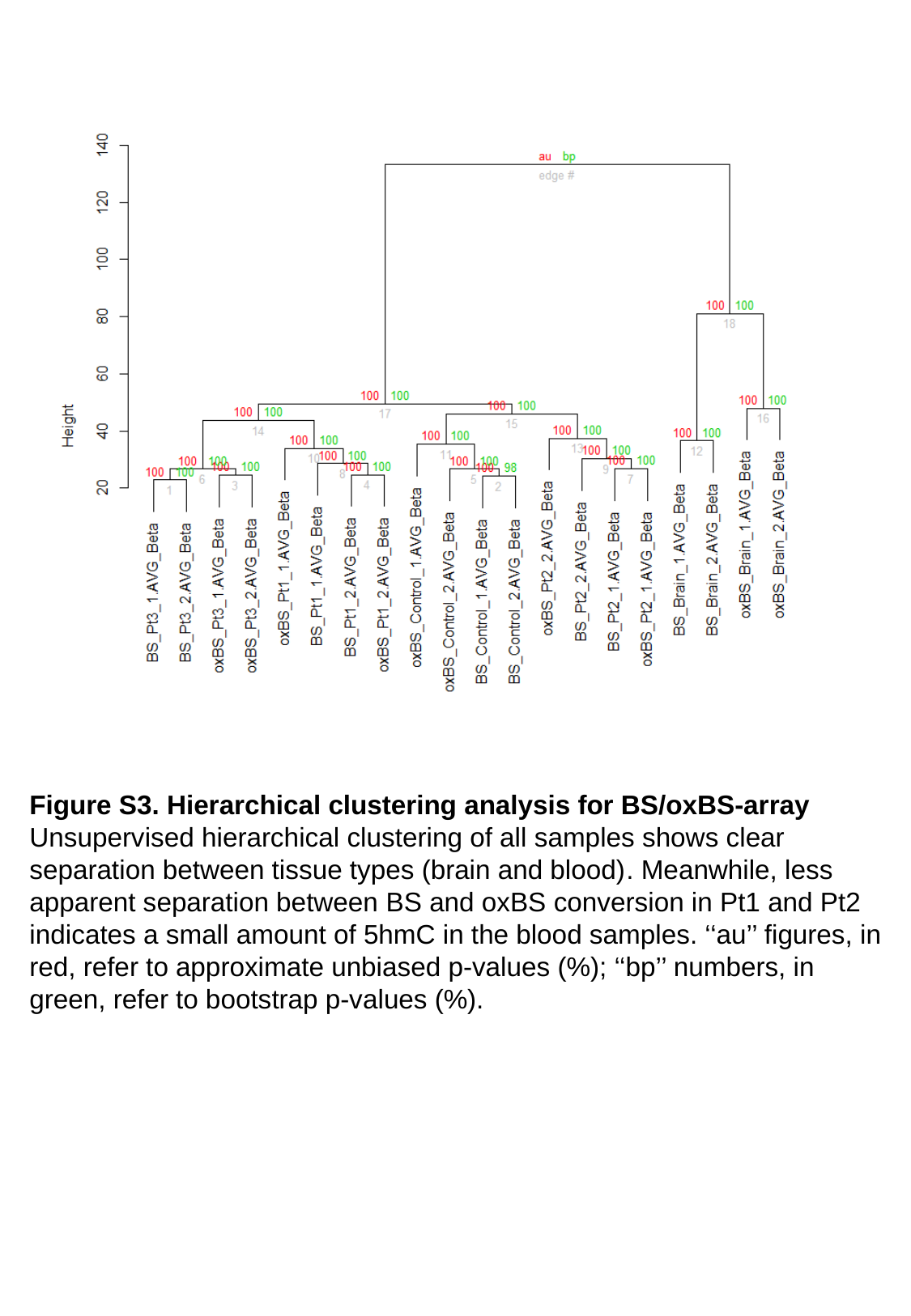

Figure S3. Hierarchical clustering analysis for BS/oxBS-array
Unsupervised hierarchical clustering of all samples shows clear separation between tissue types (brain and blood). Meanwhile, less apparent separation between BS and oxBS conversion in Pt1 and Pt2 indicates a small amount of 5hmC in the blood samples. ‘‘au’’ figures, in red, refer to approximate unbiased p-values (%); ‘‘bp’’ numbers, in green, refer to bootstrap p-values (%).
