# Supplementary material for: Exploration of hydroxymethylation in Kagami-Ogata syndrome caused by hypermethylation of imprinting control regions
Source: Clin Epigenetics. 2015 Aug 28;7(1):90. doi: 10.1186/s13148-015-0124-y (PMC4552283; doi:10.1186/s13148-015-0124-y)
Supplement: Additional file 6: Figure S4. — Methylation/hydroxymethylation analysis at the MEG3-DMR, PLAGL1-DMR, PEG10-DMR, MEST-DMR, H19-DMR, KvDMR1, SNRPN-DMR, and GNAS exon A/B-DMR by BS/oxBS-array. (PPB 240 kb) [file 13148_2015_124_MOESM6_ESM.pptx]

## Slide 1
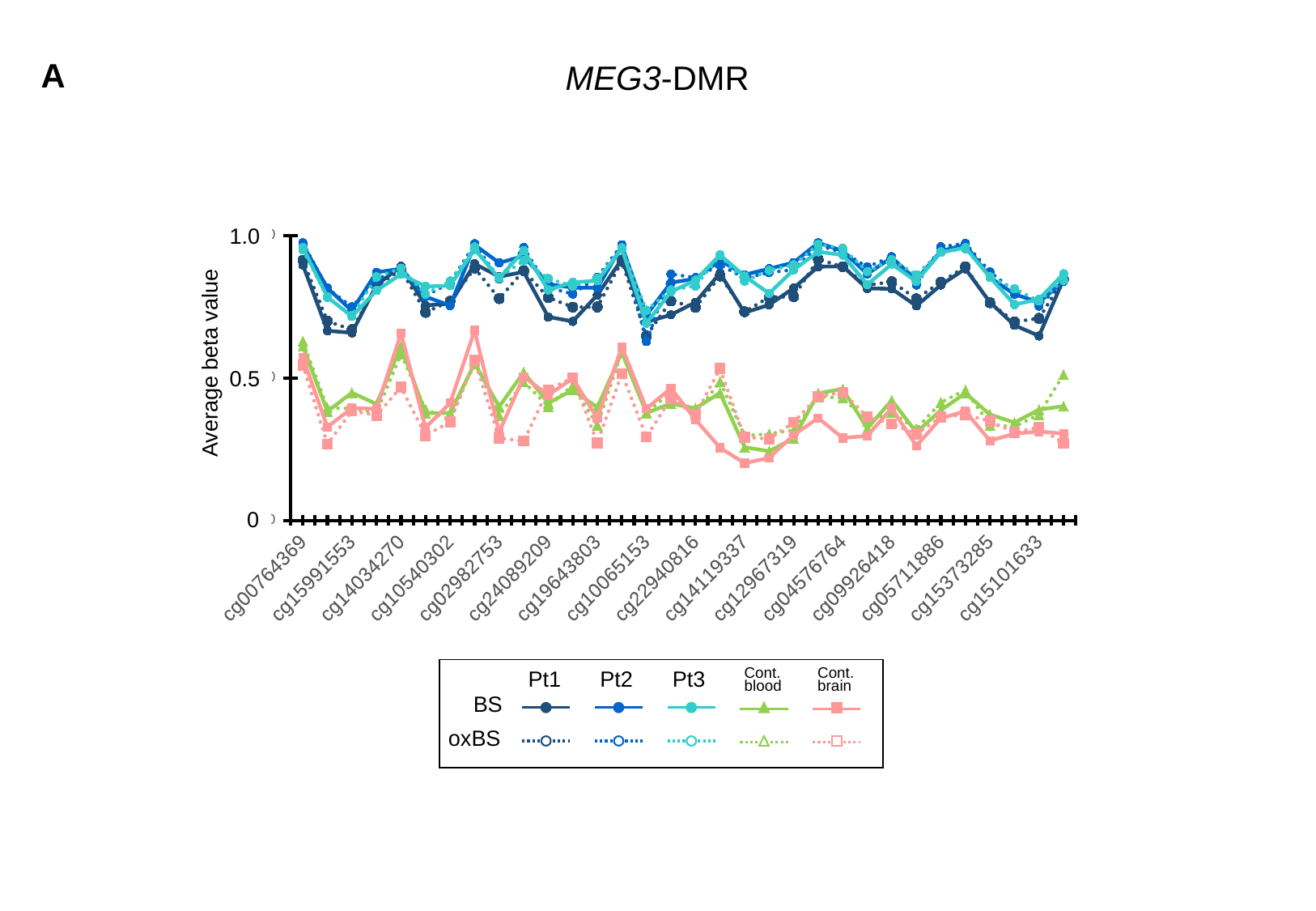

A
MEG3-DMR
1.0
### Chart
| Category | BS (Pt 1) | oxBS (Pt 1) | BS (Pt 2) | oxBS (Pt 2) | BS (Pt 3) | oxBS (Pt 3) | BS (Cont) | oxBS (Cont) | BS (Brain) | oxBS (Brain) |
|---|---|---|---|---|---|---|---|---|---|---|
| cg00764369 | 0.896135 | 0.9127513 | 0.96596385 | 0.9736792 | 0.95721135 | 0.9485409 | 0.6107707 | 0.62808095 | 0.5704127 | 0.54400225 |
| cg10515315 | 0.66507235 | 0.69887505 | 0.8152777 | 0.813905 | 0.7835663 | 0.7825039 | 0.3824322 | 0.39505745 | 0.327652 | 0.2676784 |
| cg15991553 | 0.65801125 | 0.66873 | 0.73497905 | 0.7488907 | 0.7154104 | 0.7184989 | 0.44789655 | 0.3919024 | 0.3938575 | 0.38378885 |
| cg23870378 | 0.82531685 | 0.82152225 | 0.87030865 | 0.8441735 | 0.8062572 | 0.8539833 | 0.40804995 | 0.37790115 | 0.39133685 | 0.36848365 |
| cg14034270 | 0.89115965 | 0.87961535 | 0.8826358 | 0.8849502 | 0.86337145 | 0.88389475 | 0.6109297 | 0.5833208 | 0.6564845 | 0.4680197 |
| cg16567044 | 0.75426975 | 0.7296537 | 0.7849276 | 0.7879837 | 0.8204738 | 0.7938443 | 0.37578775 | 0.38924775 | 0.32509265 | 0.29780605 |
| cg10540302 | 0.7601147 | 0.76867255 | 0.7535845 | 0.8332668 | 0.82341625 | 0.8394771 | 0.37702105 | 0.3510109 | 0.4121063 | 0.34548525 |
| cg02313552 | 0.89995345 | 0.8848814 | 0.9660813 | 0.9701293 | 0.95043455 | 0.95901325 | 0.5503575 | 0.55728575 | 0.6659634 | 0.5615594 |
| cg02982753 | 0.85498535 | 0.77799425 | 0.9035069 | 0.84642495 | 0.8493092 | 0.85199785 | 0.39858895 | 0.3677872 | 0.3100528 | 0.28859585 |
| cg02888166 | 0.87164825 | 0.8759693 | 0.92747455 | 0.95691045 | 0.94594685 | 0.91069765 | 0.5199197 | 0.4860276 | 0.50371395 | 0.27878215 |
| cg24089209 | 0.71350275 | 0.78189455 | 0.82863835 | 0.81679435 | 0.80841315 | 0.8485341 | 0.4114067 | 0.39976145 | 0.4394673 | 0.4582338 |
| cg16738180 | 0.69816595 | 0.74772575 | 0.81661795 | 0.79399975 | 0.83524025 | 0.8220696 | 0.45773575 | 0.4740007 | 0.49831295 | 0.5007204 |
| cg19643803 | 0.79032935 | 0.74843 | 0.81579815 | 0.85322815 | 0.8403517 | 0.84925325 | 0.3960306 | 0.33405445 | 0.3585705 | 0.2713753 |
| cg10943497 | 0.91614375 | 0.90843435 | 0.9629377 | 0.9667309 | 0.9527681 | 0.95588475 | 0.5895963 | 0.5907302 | 0.60887495 | 0.51517505 |
| cg10065153 | 0.6955497 | 0.6467838 | 0.72031805 | 0.62772635 | 0.6919762 | 0.7371539 | 0.3767715 | 0.37881705 | 0.3915626 | 0.29250915 |
| cg09280976 | 0.72111125 | 0.76976005 | 0.83378945 | 0.8639712 | 0.80217675 | 0.8080086 | 0.409878 | 0.4478729 | 0.4639261 | 0.42846565 |
| cg22940816 | 0.7651332 | 0.7471354 | 0.8478444 | 0.85154215 | 0.8422326 | 0.8223272 | 0.3933383 | 0.3723679 | 0.35353995 | 0.3705788 |
| cg14070323 | 0.8671583 | 0.8573276 | 0.91224345 | 0.8962918 | 0.93150205 | 0.9295158 | 0.4470745 | 0.48738395 | 0.2550116 | 0.5322464 |
| cg14119337 | 0.7284674 | 0.7318578 | 0.8616508 | 0.85016815 | 0.8569028 | 0.83921725 | 0.25664335 | 0.2967987 | 0.2005656 | 0.29204615 |
| cg20952167 | 0.75609565 | 0.78279145 | 0.8826242 | 0.86963635 | 0.7962745 | 0.87690415 | 0.24279035 | 0.30371035 | 0.21948725 | 0.2852203 |
| cg12967319 | 0.81471885 | 0.78507235 | 0.90439335 | 0.8792015 | 0.87886715 | 0.89649915 | 0.2877189 | 0.31701405 | 0.2992478 | 0.3435834 |
| cg04304932 | 0.8900348 | 0.9156321 | 0.97364665 | 0.96400855 | 0.94229515 | 0.9676649 | 0.44589095 | 0.4341622 | 0.3591242 | 0.43537825 |
| cg04576764 | 0.8908228 | 0.8898095 | 0.9454754 | 0.9410324 | 0.93096105 | 0.9545635 | 0.4608624 | 0.4293449 | 0.28898705 | 0.4490314 |
| cg25836301 | 0.81342045 | 0.82472975 | 0.8644898 | 0.8892592 | 0.82746355 | 0.87278255 | 0.32270625 | 0.34722505 | 0.29647085 | 0.36347195 |
| cg09926418 | 0.81244575 | 0.83722035 | 0.9219127 | 0.9245694 | 0.8989276 | 0.9144605 | 0.4212793 | 0.3780144 | 0.3939771 | 0.3384296 |
| cg23176399 | 0.75224395 | 0.77724365 | 0.84371965 | 0.8258463 | 0.8383036 | 0.8617587 | 0.3086818 | 0.3204529 | 0.2611969 | 0.30232295 |
| cg05711886 | 0.8256007 | 0.83563165 | 0.94454415 | 0.95944405 | 0.94002315 | 0.941946 | 0.3862915 | 0.41374385 | 0.3581589 | 0.3620754 |
| cg15419911 | 0.8825991 | 0.88972565 | 0.9670613 | 0.97142445 | 0.95678145 | 0.95269845 | 0.44612385 | 0.45711825 | 0.38288745 | 0.3711782 |
| cg15373285 | 0.76525065 | 0.7636021 | 0.85207795 | 0.87161195 | 0.85313635 | 0.8543157 | 0.3713742 | 0.33452275 | 0.27943395 | 0.3480694 |
| cg14123427 | 0.6844121 | 0.6965759 | 0.79317985 | 0.8032494 | 0.75722535 | 0.8126917 | 0.3432235 | 0.32904975 | 0.30417315 | 0.30906145 |
| cg15101633 | 0.6471028 | 0.7085157 | 0.76481185 | 0.7519722 | 0.7760434 | 0.7694348 | 0.388595 | 0.3715009 | 0.31174555 | 0.3269014 |
| cg26374305 | 0.84581595 | 0.8461227 | 0.8390988 | 0.83936515 | 0.8649926 | 0.8423345 | 0.40044575 | 0.51188495 | 0.30422135 | 0.26984315 |Average beta value
0.5
0
Pt1
Pt2
Pt3
Cont.
blood
BS
oxBS
Cont.
brain

## Slide 2
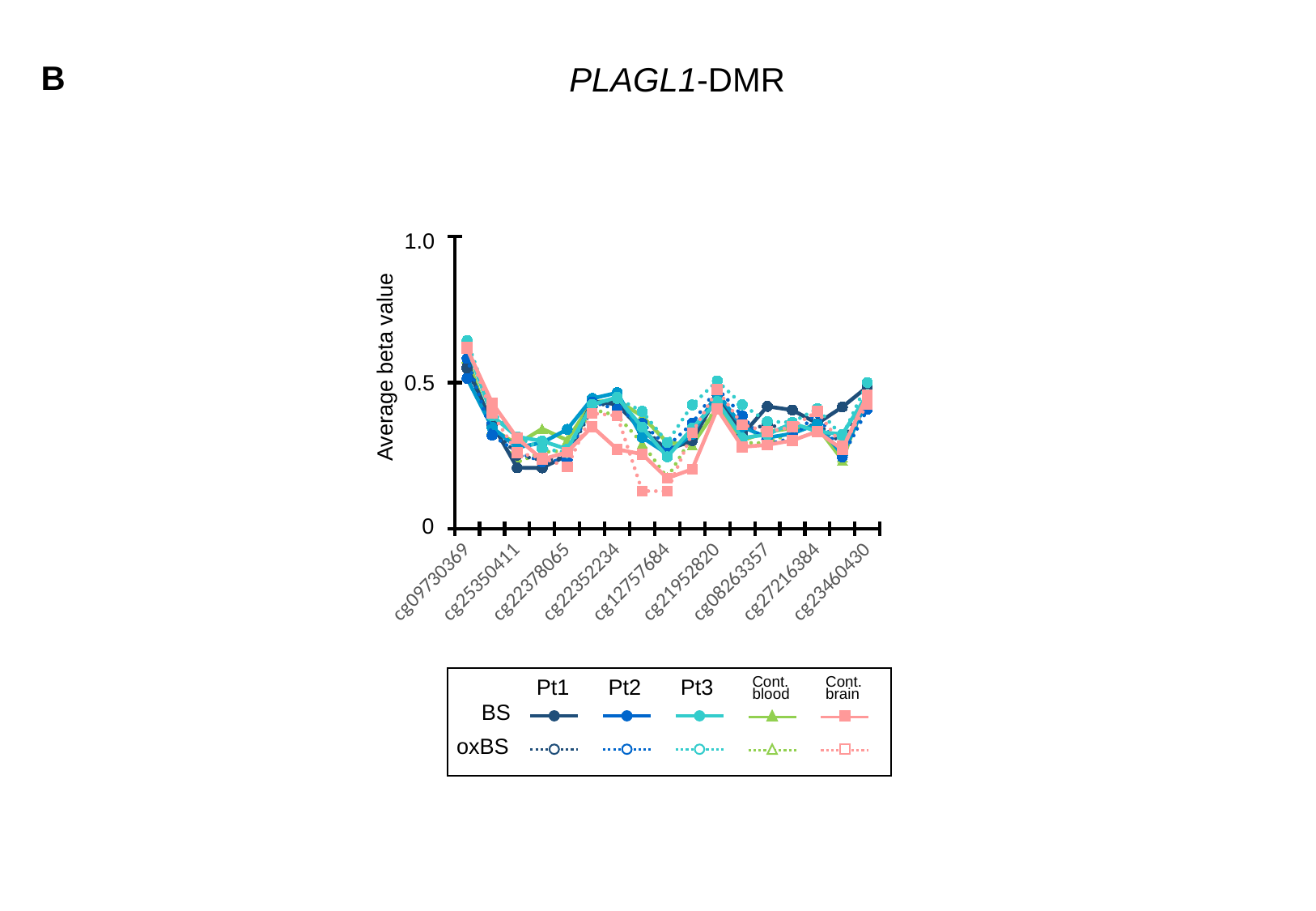

B
PLAGL1-DMR
### Chart
| Category | BS_pool | OX_pool | BS_869 | OX_869 | BS_3410 | OX_3410 | BS_5152 | OX_5152 | BS_Brain | OX_Brain |
|---|---|---|---|---|---|---|---|---|---|---|
| cg09730369 | 0.58473725 | 0.58598 | 0.55420395 | 0.5479697 | 0.51430595 | 0.58231235 | 0.62498635 | 0.6443174 | 0.6158492 | 0.62229455 |
| cg17865602 | 0.34118505 | 0.3403994 | 0.35940155 | 0.34842875 | 0.34644455 | 0.3196748 | 0.38622885 | 0.4003389 | 0.4307133 | 0.39507325 |
| cg25350411 | 0.2891073 | 0.22667335 | 0.20817755 | 0.2518078 | 0.2774592 | 0.2614371 | 0.31428275 | 0.3119257 | 0.30998545 | 0.2592293 |
| cg07077459 | 0.3418078 | 0.2612344 | 0.2076981 | 0.2338064 | 0.294009 | 0.22894475 | 0.2995098 | 0.27374965 | 0.236627 | 0.24174725 |
| cg22378065 | 0.3027146 | 0.26759405 | 0.25024405 | 0.24319795 | 0.33954575 | 0.23550795 | 0.27199235 | 0.26027335 | 0.26175425 | 0.2112981 |
| cg10007452 | 0.43332715 | 0.4086629 | 0.4351632 | 0.41285855 | 0.4455447 | 0.43348305 | 0.42457645 | 0.4253922 | 0.34880915 | 0.3948283 |
| cg22352234 | 0.4397772 | 0.38669915 | 0.4254786 | 0.4401587 | 0.46524695 | 0.40820325 | 0.44976235 | 0.44570265 | 0.2714521 | 0.3865274 |
| cg00702231 | 0.384774 | 0.29206095 | 0.3346567 | 0.351358 | 0.31240595 | 0.36044345 | 0.34693985 | 0.40130035 | 0.2544443 | 0.12822655 |
| cg12757684 | 0.2942884 | 0.17374705 | 0.269326 | 0.26613845 | 0.25512575 | 0.28126065 | 0.2454321 | 0.2947844 | 0.17240305 | 0.12821055 |
| cg21526238 | 0.28653615 | 0.3150585 | 0.30090295 | 0.30876465 | 0.32345485 | 0.36037505 | 0.3438293 | 0.4235085 | 0.2032365 | 0.3281034 |
| cg21952820 | 0.41334165 | 0.41105805 | 0.45523345 | 0.4724786 | 0.4643073 | 0.4807674 | 0.43532315 | 0.50592775 | 0.40930215 | 0.4762281 |
| cg05326984 | 0.3018873 | 0.2936691 | 0.32044955 | 0.3324548 | 0.3487656 | 0.38548175 | 0.3100922 | 0.4241626 | 0.2797204 | 0.35597245 |
| cg08263357 | 0.33140635 | 0.29266295 | 0.41844765 | 0.3561762 | 0.3109702 | 0.2886923 | 0.3219905 | 0.3658686 | 0.28654125 | 0.3312826 |
| cg11532302 | 0.34351135 | 0.3287173 | 0.4060395 | 0.33593695 | 0.32504535 | 0.3097083 | 0.3608557 | 0.36387075 | 0.30160695 | 0.3479117 |
| cg27216384 | 0.34778895 | 0.35659135 | 0.35978485 | 0.35926495 | 0.35810195 | 0.39794955 | 0.3322381 | 0.41086375 | 0.33252705 | 0.4020256 |
| cg17895149 | 0.23469315 | 0.24560675 | 0.416333 | 0.2895725 | 0.2520286 | 0.24518 | 0.32273005 | 0.31551295 | 0.2709266 | 0.28197705 |
| cg23460430 | 0.46391075 | 0.43120325 | 0.4856279 | 0.46846075 | 0.4417455 | 0.40896935 | 0.45969735 | 0.5002509 | 0.4574115 | 0.42576465 |1.0
Average beta value
0.5
0
Pt1
Pt2
Pt3
Cont.
blood
BS
oxBS
Cont.
brain

## Slide 3
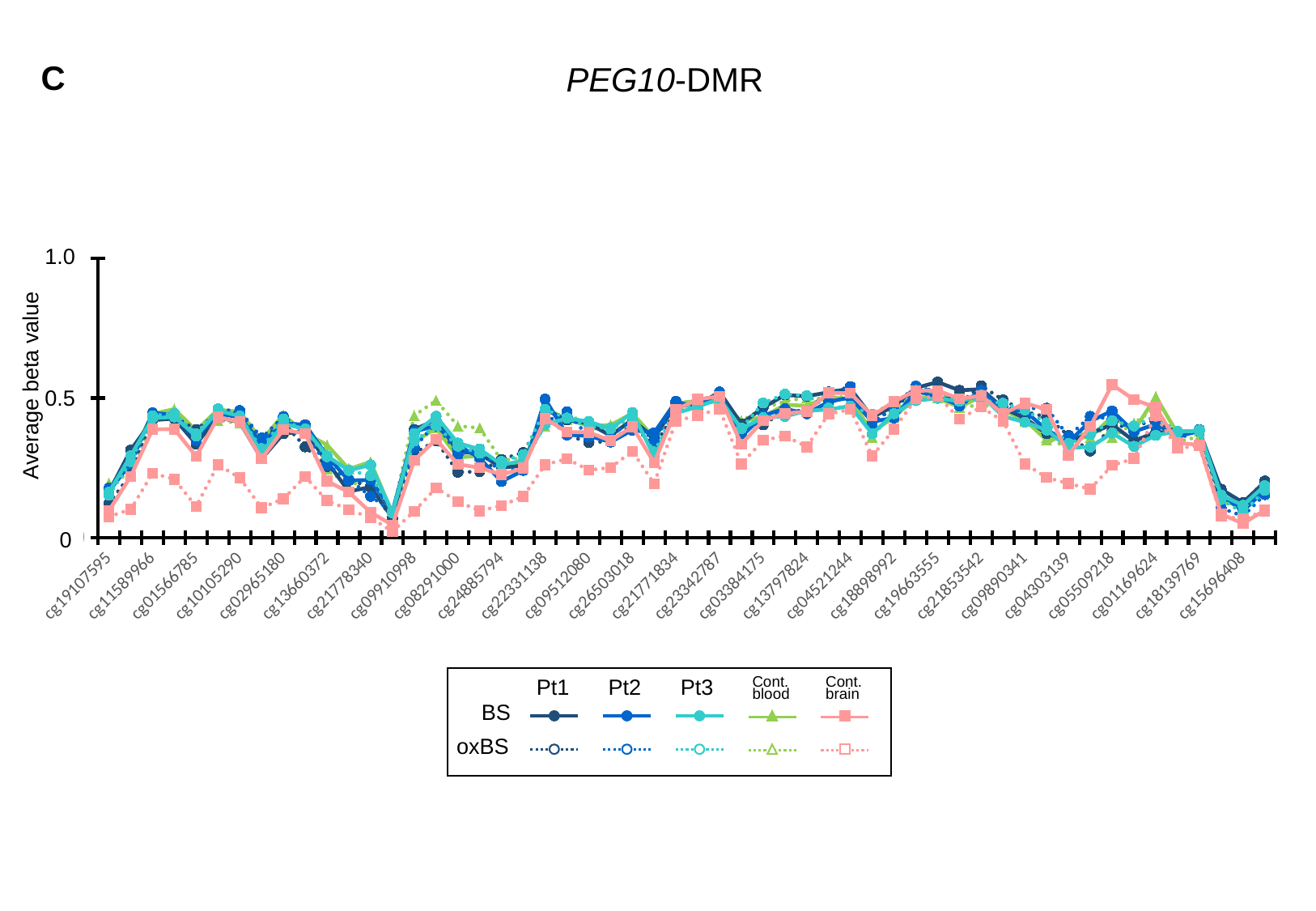

C
PEG10-DMR
### Chart
| Category | BS_pool | OX_pool | BS_869 | OX_869 | BS_3410 | OX_3410 | BS_5152 | OX_5152 | BS_Brain | OX_Brain |
|---|---|---|---|---|---|---|---|---|---|---|
| cg19107595 | 0.1726372 | 0.19498315 | 0.16269465 | 0.1221034 | 0.16547445 | 0.176382115 | 0.16239155 | 0.1522053 | 0.09604624 | 0.07458837 |
| cg19924104 | 0.2862165 | 0.2296758 | 0.31349035 | 0.2262139 | 0.26638235 | 0.26191955 | 0.29258245 | 0.27218915 | 0.2189725 | 0.10068924 |
| cg11589966 | 0.44304335 | 0.420416 | 0.42071515 | 0.43088705 | 0.4465376 | 0.44076745 | 0.427844 | 0.43675385 | 0.3875987 | 0.2305034 |
| cg27435646 | 0.4605031 | 0.4331848 | 0.42664865 | 0.43273615 | 0.44048565 | 0.43346015 | 0.43248335 | 0.44550725 | 0.3884493 | 0.2086423 |
| cg01566785 | 0.38355315 | 0.3395608 | 0.3353816 | 0.38651415 | 0.35719865 | 0.34812965 | 0.36362335 | 0.3717227 | 0.29077715 | 0.110966635 |
| cg05819130 | 0.45851215 | 0.4193484 | 0.431416 | 0.44610575 | 0.44488745 | 0.45720875 | 0.45381865 | 0.46138805 | 0.43117765 | 0.26103635 |
| cg10105290 | 0.45100385 | 0.410579 | 0.4131071 | 0.44818805 | 0.4308773 | 0.45493005 | 0.43605875 | 0.43497305 | 0.414615 | 0.21345205 |
| cg19079047 | 0.3480238 | 0.30563895 | 0.2821876 | 0.35079365 | 0.3380118 | 0.3565659 | 0.30288275 | 0.3183332 | 0.28232005 | 0.106469255 |
| cg02965180 | 0.4398048 | 0.37738885 | 0.37246925 | 0.41167205 | 0.4089511 | 0.4335737 | 0.4072166 | 0.4250857 | 0.3864789 | 0.138641015 |
| cg05277165 | 0.373881 | 0.4012105 | 0.38049265 | 0.32497635 | 0.4044442 | 0.37462775 | 0.3891916 | 0.38086245 | 0.37247365 | 0.2180922 |
| cg13660372 | 0.33164685 | 0.24498925 | 0.2719261 | 0.25453565 | 0.292168 | 0.25940755 | 0.29225885 | 0.2899523 | 0.20194915 | 0.13248907 |
| cg06674062 | 0.245355 | 0.1844018 | 0.16504465 | 0.206152 | 0.2043929 | 0.20814475 | 0.2405017 | 0.24097565 | 0.16262645 | 0.09944756 |
| cg21778340 | 0.27124785 | 0.2149874 | 0.18099505 | 0.18272105 | 0.20624165 | 0.1476625 | 0.26007815 | 0.22186065 | 0.090333875 | 0.07081178 |
| cg07921535 | 0.08628956 | 0.085467285 | 0.070000005 | 0.063784445 | 0.09367862 | 0.092135815 | 0.088352775 | 0.09410045 | 0.0446398 | 0.02144688 |
| cg09910998 | 0.3421298 | 0.43700305 | 0.38722885 | 0.30163155 | 0.38007815 | 0.32238915 | 0.3722188 | 0.3459112 | 0.27708695 | 0.094169 |
| cg27393730 | 0.392268 | 0.4912898 | 0.4214121 | 0.34727005 | 0.4003278 | 0.41101495 | 0.4347652 | 0.4007093 | 0.3516775 | 0.17768785 |
| cg08291000 | 0.28720565 | 0.39996405 | 0.30836445 | 0.23414585 | 0.33137865 | 0.2963375 | 0.33846625 | 0.3275387 | 0.26223895 | 0.1287067 |
| cg27230044 | 0.29262565 | 0.3924739 | 0.30120215 | 0.2363686 | 0.28471675 | 0.3174658 | 0.31664545 | 0.30211365 | 0.25036785 | 0.095372265 |
| cg24885794 | 0.26115165 | 0.2763875 | 0.2499506 | 0.279944 | 0.200865 | 0.2283754 | 0.26152875 | 0.2727601 | 0.22502215 | 0.11428815 |
| cg26997085 | 0.26469855 | 0.2853752 | 0.25845755 | 0.30487205 | 0.24051625 | 0.25557035 | 0.2739076 | 0.29662785 | 0.24886635 | 0.14700115 |
| cg22331138 | 0.4638664 | 0.3977579 | 0.4591368 | 0.44133295 | 0.4961194 | 0.41469795 | 0.40488125 | 0.45961585 | 0.42646555 | 0.26119065 |
| cg16492735 | 0.42272235 | 0.45078995 | 0.42134635 | 0.3673273 | 0.36812315 | 0.45081475 | 0.42761035 | 0.42852505 | 0.37796815 | 0.2819446 |
| cg09512080 | 0.40094365 | 0.39281825 | 0.4084163 | 0.3405872 | 0.3640297 | 0.36005625 | 0.4158937 | 0.3966432 | 0.37727395 | 0.24174965 |
| cg00906934 | 0.4029965 | 0.3847098 | 0.36536195 | 0.3447248 | 0.34216135 | 0.3634129 | 0.3890263 | 0.3922953 | 0.3469208 | 0.2504408 |
| cg26503018 | 0.4451232 | 0.4374639 | 0.42254715 | 0.40476435 | 0.3847164 | 0.4216095 | 0.44854365 | 0.4331904 | 0.3975564 | 0.3090201 |
| cg27120649 | 0.3572488 | 0.34475345 | 0.35656075 | 0.32678585 | 0.37569965 | 0.3458826 | 0.2915322 | 0.3069935 | 0.26779535 | 0.19273495 |
| cg21771834 | 0.4804127 | 0.4746541 | 0.4787269 | 0.45374815 | 0.48824625 | 0.47773935 | 0.445994 | 0.45892625 | 0.4586366 | 0.41785295 |
| cg27001184 | 0.4902928 | 0.49674845 | 0.47471355 | 0.49668955 | 0.46303455 | 0.4805284 | 0.46902065 | 0.473787 | 0.4952365 | 0.43777315 |
| cg23342787 | 0.4890336 | 0.4734483 | 0.52154225 | 0.5032868 | 0.5216797 | 0.513171 | 0.49756635 | 0.4912667 | 0.50636905 | 0.46119485 |
| cg14873490 | 0.41764925 | 0.41798985 | 0.40763995 | 0.39195605 | 0.3624136 | 0.3738184 | 0.3904136 | 0.39174325 | 0.3350979 | 0.26336885 |
| cg03384175 | 0.4319967 | 0.4628739 | 0.4661146 | 0.4038591 | 0.4374489 | 0.41931405 | 0.4295704 | 0.48212085 | 0.4189132 | 0.3483902 |
| cg11985632 | 0.47623425 | 0.48860855 | 0.51117205 | 0.45627465 | 0.46151935 | 0.45514865 | 0.43439485 | 0.51393325 | 0.44478405 | 0.363479 |
| cg13797824 | 0.4720551 | 0.50095525 | 0.5065387 | 0.45321605 | 0.4440097 | 0.4514484 | 0.45444735 | 0.50834665 | 0.451685 | 0.32511765 |
| cg11175683 | 0.50520615 | 0.4959964 | 0.5209321 | 0.52161165 | 0.4862313 | 0.51893645 | 0.4591714 | 0.4645571 | 0.51900785 | 0.44285925 |
| cg04521244 | 0.4930719 | 0.5089357 | 0.53381165 | 0.53241565 | 0.5027898 | 0.5412824 | 0.4713384 | 0.4697983 | 0.5173988 | 0.4594844 |
| cg11562309 | 0.37862115 | 0.3599596 | 0.4316151 | 0.41308375 | 0.4151144 | 0.4012762 | 0.37190755 | 0.44178245 | 0.4370601 | 0.2921407 |
| cg18898992 | 0.4310278 | 0.4572221 | 0.47099135 | 0.4668798 | 0.42913825 | 0.47302975 | 0.44418275 | 0.4862089 | 0.48755245 | 0.3884066 |
| cg05096321 | 0.50341515 | 0.52621165 | 0.53637405 | 0.52652125 | 0.52490755 | 0.5427675 | 0.4907778 | 0.5270914 | 0.52637515 | 0.4971363 |
| cg19663555 | 0.4998411 | 0.5230652 | 0.5574681 | 0.53203995 | 0.5051779 | 0.5192514 | 0.4994177 | 0.52076555 | 0.5268866 | 0.50785755 |
| cg06695761 | 0.4673185 | 0.4670663 | 0.52759655 | 0.48021495 | 0.4748314 | 0.47158375 | 0.4880691 | 0.4974611 | 0.4978854 | 0.4250069 |
| cg21853542 | 0.5063994 | 0.4748904 | 0.5320966 | 0.54444535 | 0.5272477 | 0.5196642 | 0.5105385 | 0.4922572 | 0.5083403 | 0.46759715 |
| cg04634483 | 0.45614475 | 0.4636115 | 0.4640082 | 0.49319335 | 0.45665345 | 0.4624112 | 0.43465735 | 0.4812242 | 0.44344695 | 0.41709825 |
| cg09890341 | 0.4215307 | 0.41795795 | 0.4283148 | 0.45160595 | 0.4538615 | 0.46275345 | 0.41363195 | 0.4577022 | 0.48188905 | 0.26417045 |
| cg20041873 | 0.34895865 | 0.3589147 | 0.3721228 | 0.4316795 | 0.38583745 | 0.4636129 | 0.38509025 | 0.41103905 | 0.4592897 | 0.21488405 |
| cg04303139 | 0.3599317 | 0.3196839 | 0.3466595 | 0.3654874 | 0.34179845 | 0.3625921 | 0.3218878 | 0.3368708 | 0.2953993 | 0.193345 |
| cg03436478 | 0.35926325 | 0.4008505 | 0.3724113 | 0.3099599 | 0.417248 | 0.43421445 | 0.32430115 | 0.36883035 | 0.39871585 | 0.17412385 |
| cg05509218 | 0.4339113 | 0.3584317 | 0.40531125 | 0.399256 | 0.4538144 | 0.42538205 | 0.37454135 | 0.41836455 | 0.5479477 | 0.2584405 |
| cg22924867 | 0.38225535 | 0.4097609 | 0.3459241 | 0.3992733 | 0.3793208 | 0.3830116 | 0.32663395 | 0.3997164 | 0.4946813 | 0.28321475 |
| cg01169624 | 0.50483945 | 0.4649405 | 0.3777374 | 0.42132325 | 0.4063443 | 0.40109115 | 0.36645225 | 0.43507405 | 0.46665765 | 0.4372324 |
| cg03682823 | 0.37260425 | 0.34364855 | 0.37712095 | 0.3730974 | 0.3631035 | 0.3516142 | 0.3807461 | 0.3771881 | 0.33933355 | 0.31978495 |
| cg18139769 | 0.3815767 | 0.361447 | 0.3822784 | 0.38137105 | 0.3810236 | 0.386883 | 0.38161835 | 0.38495145 | 0.3317619 | 0.328608 |
| cg12397924 | 0.13605315 | 0.14857395 | 0.1728579 | 0.13644845 | 0.1538529 | 0.10540169 | 0.15141545 | 0.1363145 | 0.08391819 | 0.07623223 |
| cg15696408 | 0.10556805 | 0.10125577 | 0.12545645 | 0.1210706 | 0.094465505 | 0.076715725 | 0.11539215 | 0.08885081 | 0.049891785 | 0.067274525 |
| cg22820921 | 0.1777702 | 0.1940517 | 0.19930235 | 0.20261215 | 0.169967 | 0.153868 | 0.18554815 | 0.16904895 | 0.09805118 | 0.094628195 |1.0
Average beta value
0.5
0
Pt1
Pt2
Pt3
Cont.
blood
BS
oxBS
Cont.
brain

## Slide 4
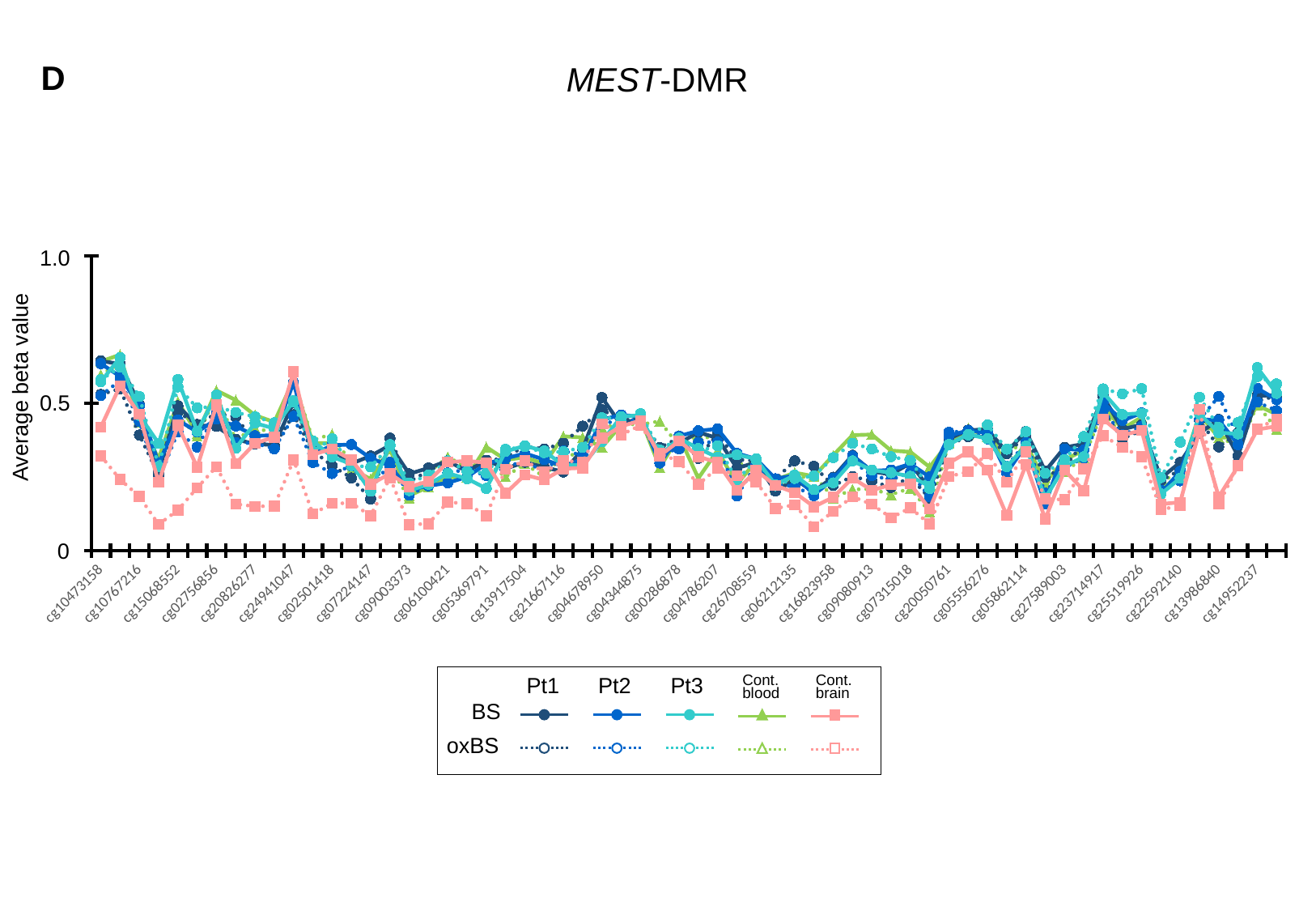

D
MEST-DMR
### Chart
| Category | BS_pool | OX_pool | BS_869 | OX_869 | BS_3410 | OX_3410 | BS_5152 | OX_5152 | BS_Brain | OX_Brain |
|---|---|---|---|---|---|---|---|---|---|---|
| cg10473158 | 0.6415335 | 0.5967036 | 0.6437867 | 0.53012835 | 0.634626 | 0.52666825 | 0.5729548 | 0.5795152 | 0.4186514 | 0.3215098 |
| cg05222671 | 0.6647381 | 0.63477895 | 0.63300965 | 0.5492214 | 0.5913047 | 0.5767713 | 0.6548768 | 0.6225579 | 0.5591012 | 0.24196035 |
| cg10767216 | 0.4370579 | 0.44570295 | 0.4965937 | 0.39230805 | 0.49191195 | 0.43868305 | 0.45891195 | 0.5229186 | 0.462617 | 0.18339535 |
| cg12070022 | 0.31236205 | 0.3106876 | 0.26765175 | 0.25606575 | 0.29478285 | 0.23880255 | 0.3636677 | 0.28544875 | 0.2316542 | 0.088857385 |
| cg15068552 | 0.5053535 | 0.4579635 | 0.4913243 | 0.47048115 | 0.44359255 | 0.4026458 | 0.580677 | 0.55550995 | 0.42546415 | 0.1374557 |
| cg17347253 | 0.39641765 | 0.38847785 | 0.42519275 | 0.42821645 | 0.40236025 | 0.351173 | 0.40437725 | 0.48429045 | 0.28284795 | 0.2131057 |
| cg02756856 | 0.5434839 | 0.52518165 | 0.42122835 | 0.4656562 | 0.44621765 | 0.46832815 | 0.52838235 | 0.48908885 | 0.49415945 | 0.2849264 |
| cg26275543 | 0.5107575 | 0.37692075 | 0.3778423 | 0.4540231 | 0.42268255 | 0.3665902 | 0.34716775 | 0.468974 | 0.295391 | 0.157729705 |
| cg20826277 | 0.45922405 | 0.41605735 | 0.3614359 | 0.36722795 | 0.38975575 | 0.36415395 | 0.432661 | 0.45425555 | 0.36420115 | 0.1503319 |
| cg14584935 | 0.4361631 | 0.39948365 | 0.35850635 | 0.3560546 | 0.39110635 | 0.3456785 | 0.4141653 | 0.4339667 | 0.3848308 | 0.1512646 |
| cg24941047 | 0.5789774 | 0.5820408 | 0.50307425 | 0.4609696 | 0.5706548 | 0.45590135 | 0.5084912 | 0.50393495 | 0.60667805 | 0.30807935 |
| cg07427065 | 0.3644496 | 0.3750791 | 0.3455781 | 0.3386003 | 0.3252391 | 0.2988796 | 0.3718774 | 0.3366703 | 0.32596115 | 0.12484825 |
| cg02501418 | 0.36436695 | 0.3954141 | 0.34123935 | 0.2880368 | 0.35763875 | 0.26152775 | 0.31800775 | 0.3795464 | 0.3449101 | 0.1601059 |
| cg08229366 | 0.27655645 | 0.29341635 | 0.29512845 | 0.2464578 | 0.35993005 | 0.2872403 | 0.29393155 | 0.28860695 | 0.30789395 | 0.1612211 |
| cg07224147 | 0.2429819 | 0.3225615 | 0.32120965 | 0.17513365 | 0.313072 | 0.20362995 | 0.20162775 | 0.2838373 | 0.22481605 | 0.118636625 |
| cg27417677 | 0.3444664 | 0.28580085 | 0.35733975 | 0.38153555 | 0.2882487 | 0.2996312 | 0.3596569 | 0.35530205 | 0.2612363 | 0.24678885 |
| cg09003373 | 0.1865507 | 0.1776302 | 0.2604865 | 0.23417465 | 0.2053846 | 0.1875318 | 0.2035786 | 0.22980735 | 0.2163418 | 0.08838998 |
| cg14088957 | 0.21594075 | 0.26550775 | 0.28013395 | 0.27036475 | 0.219938 | 0.2219027 | 0.22467595 | 0.2564342 | 0.23510385 | 0.091180725 |
| cg06100421 | 0.24861835 | 0.3172417 | 0.30479705 | 0.3037684 | 0.22993895 | 0.2633474 | 0.26256575 | 0.3045171 | 0.30018715 | 0.1650937 |
| cg20297423 | 0.2502493 | 0.28318665 | 0.26817935 | 0.2743369 | 0.250066 | 0.27583675 | 0.24397295 | 0.26910325 | 0.30440245 | 0.15878655 |
| cg05369791 | 0.35145235 | 0.2966906 | 0.3068701 | 0.3016626 | 0.29486465 | 0.25454845 | 0.2110646 | 0.2629151 | 0.29821435 | 0.1184145 |
| cg08734637 | 0.30827675 | 0.2508262 | 0.27848035 | 0.31707025 | 0.3129668 | 0.28088775 | 0.3366259 | 0.3435464 | 0.19434265 | 0.27648755 |
| cg13917504 | 0.3161206 | 0.2949392 | 0.29984025 | 0.35448085 | 0.3297642 | 0.3076695 | 0.35559895 | 0.3523748 | 0.25690075 | 0.30549345 |
| cg25407198 | 0.2942675 | 0.2499796 | 0.28329305 | 0.34422265 | 0.30914555 | 0.26310005 | 0.33786615 | 0.3324899 | 0.2401183 | 0.26625285 |
| cg21667116 | 0.3878341 | 0.3282476 | 0.26688615 | 0.3645919 | 0.2855941 | 0.33787635 | 0.2893958 | 0.3356725 | 0.277888 | 0.30444435 |
| cg23156962 | 0.38388415 | 0.32162665 | 0.3437442 | 0.42262705 | 0.31033435 | 0.32409665 | 0.2898337 | 0.3501732 | 0.2807012 | 0.3013474 |
| cg04678950 | 0.3505084 | 0.4171093 | 0.5196329 | 0.4781942 | 0.4445484 | 0.433153 | 0.37005405 | 0.45089595 | 0.38238355 | 0.4305123 |
| cg17079325 | 0.42719995 | 0.43005505 | 0.4253391 | 0.4429506 | 0.4603833 | 0.43891865 | 0.4549654 | 0.448394 | 0.42189625 | 0.39338695 |
| cg04344875 | 0.44917405 | 0.4274604 | 0.4538101 | 0.44722225 | 0.4547738 | 0.44489175 | 0.45968065 | 0.4647579 | 0.44163045 | 0.4249672 |
| cg18934293 | 0.28177175 | 0.43936525 | 0.3162902 | 0.3496 | 0.29703155 | 0.32849415 | 0.32101225 | 0.34115995 | 0.3218771 | 0.3295283 |
| cg00286878 | 0.38941785 | 0.354862 | 0.3643886 | 0.3825387 | 0.3884099 | 0.3451375 | 0.3819541 | 0.38178965 | 0.3716775 | 0.30235925 |
| cg12347392 | 0.2453348 | 0.39256255 | 0.4013444 | 0.31303845 | 0.4066411 | 0.367239 | 0.34654865 | 0.3362433 | 0.3192149 | 0.22479095 |
| cg04786207 | 0.34009275 | 0.37447985 | 0.3817191 | 0.39633345 | 0.41290205 | 0.3681712 | 0.31649305 | 0.35588885 | 0.29889065 | 0.280166 |
| cg19386484 | 0.2246143 | 0.29516775 | 0.2802228 | 0.30862435 | 0.3303352 | 0.18529255 | 0.32487855 | 0.2395188 | 0.2042233 | 0.25459405 |
| cg26708559 | 0.30418975 | 0.29151355 | 0.2985221 | 0.3050024 | 0.3108866 | 0.29256375 | 0.3115945 | 0.3076808 | 0.27182955 | 0.23401745 |
| cg22705386 | 0.24009885 | 0.2122205 | 0.20214115 | 0.2424431 | 0.2438443 | 0.22708245 | 0.2229051 | 0.2202166 | 0.2210358 | 0.14352075 |
| cg06212135 | 0.2644845 | 0.2435652 | 0.24025775 | 0.30465955 | 0.2548348 | 0.2168967 | 0.25594925 | 0.24494225 | 0.1970047 | 0.1551216 |
| cg10249538 | 0.25218535 | 0.25685005 | 0.20145885 | 0.28593245 | 0.18990635 | 0.1866081 | 0.2067368 | 0.2532614 | 0.1490504 | 0.08151788 |
| cg16823958 | 0.3232863 | 0.17906715 | 0.2492025 | 0.22104915 | 0.24211335 | 0.2456817 | 0.2300166 | 0.3155032 | 0.1811169 | 0.1333101 |
| cg27338480 | 0.3916523 | 0.20780105 | 0.3237495 | 0.2511368 | 0.3210529 | 0.30407155 | 0.3043304 | 0.36408585 | 0.2461117 | 0.18280465 |
| cg09080913 | 0.3937016 | 0.20870145 | 0.2686809 | 0.2364479 | 0.2706762 | 0.2549306 | 0.2731558 | 0.34495825 | 0.204712 | 0.1575764 |
| cg13104298 | 0.33929115 | 0.19025165 | 0.2583731 | 0.2139817 | 0.2737387 | 0.24029725 | 0.2643176 | 0.3183667 | 0.223449 | 0.1111973 |
| cg07315018 | 0.33511645 | 0.21120625 | 0.2967453 | 0.2501511 | 0.2941894 | 0.2643719 | 0.2521429 | 0.30652975 | 0.2260611 | 0.14532945 |
| cg21629528 | 0.28347035 | 0.13161675 | 0.20959775 | 0.1772186 | 0.24978085 | 0.1829202 | 0.2184621 | 0.2107111 | 0.14171215 | 0.08989045 |
| cg20050761 | 0.3673231 | 0.3651659 | 0.37973035 | 0.3601751 | 0.389697 | 0.4014134 | 0.3602463 | 0.35953605 | 0.29702075 | 0.25115895 |
| cg07870293 | 0.39564455 | 0.41473825 | 0.40884875 | 0.38678735 | 0.4077265 | 0.40593685 | 0.3942628 | 0.392724 | 0.3353383 | 0.267851 |
| cg05556276 | 0.4183478 | 0.38547905 | 0.4107987 | 0.38615805 | 0.3992782 | 0.3791784 | 0.3798993 | 0.4266966 | 0.2729404 | 0.3302361 |
| cg17580798 | 0.2782988 | 0.33685295 | 0.33507205 | 0.32920775 | 0.2634646 | 0.2858283 | 0.2851728 | 0.34391585 | 0.12017225 | 0.23149609 |
| cg05862114 | 0.36325565 | 0.36550875 | 0.4041192 | 0.3898144 | 0.35844475 | 0.3675575 | 0.35487295 | 0.40362095 | 0.292217 | 0.3343832 |
| cg01784351 | 0.2318773 | 0.22641205 | 0.27013645 | 0.2491286 | 0.15719744 | 0.19547415 | 0.1784886 | 0.2628357 | 0.107385245 | 0.17539512 |
| cg27589003 | 0.3083526 | 0.268976 | 0.35018555 | 0.28277135 | 0.34507005 | 0.2819352 | 0.28516215 | 0.30945675 | 0.27301535 | 0.17300775 |
| cg19344806 | 0.34021395 | 0.3139847 | 0.3619517 | 0.3674562 | 0.34124025 | 0.31461335 | 0.3185201 | 0.3869609 | 0.20339545 | 0.27807735 |
| cg23714917 | 0.48979955 | 0.47614345 | 0.52392445 | 0.47457155 | 0.50266795 | 0.4762575 | 0.53683235 | 0.5489433 | 0.4464742 | 0.3898951 |
| cg21200654 | 0.4128841 | 0.3850647 | 0.4116748 | 0.3799282 | 0.4411878 | 0.3926069 | 0.4620542 | 0.5319772 | 0.3916 | 0.35022895 |
| cg25519926 | 0.45291265 | 0.4234745 | 0.4278691 | 0.4073122 | 0.4671626 | 0.42102 | 0.46618445 | 0.5494191 | 0.4079121 | 0.3185013 |
| cg09462536 | 0.20632585 | 0.2299854 | 0.2462255 | 0.2377119 | 0.1950985 | 0.2050026 | 0.1921763 | 0.24777 | 0.1582814 | 0.13910935 |
| cg22592140 | 0.25768515 | 0.2740488 | 0.29941945 | 0.2733191 | 0.2691293 | 0.2368909 | 0.2457816 | 0.3677513 | 0.16392185 | 0.152507 |
| cg03588221 | 0.463609 | 0.43255835 | 0.39797595 | 0.44193995 | 0.44521475 | 0.444998 | 0.4636367 | 0.5208042 | 0.4076599 | 0.47975965 |
| cg13986840 | 0.3828852 | 0.397106 | 0.4454287 | 0.35183775 | 0.4431159 | 0.52293205 | 0.4003536 | 0.417394 | 0.1824366 | 0.15816035 |
| cg23312013 | 0.3862206 | 0.34173505 | 0.3211127 | 0.3996651 | 0.3597381 | 0.3882134 | 0.39595715 | 0.4344357 | 0.2864523 | 0.2883055 |
| cg14952237 | 0.49246405 | 0.4955969 | 0.52533525 | 0.5068724 | 0.55064555 | 0.50391825 | 0.6210368 | 0.59013825 | 0.41066945 | 0.4133228 |
| cg05260959 | 0.46268645 | 0.41218795 | 0.52760585 | 0.52578285 | 0.51231595 | 0.47479275 | 0.5334071 | 0.56591725 | 0.42221125 | 0.44714155 |1.0
Average beta value
0.5
0
Pt1
Pt2
Pt3
Cont.
blood
BS
oxBS
Cont.
brain

## Slide 5
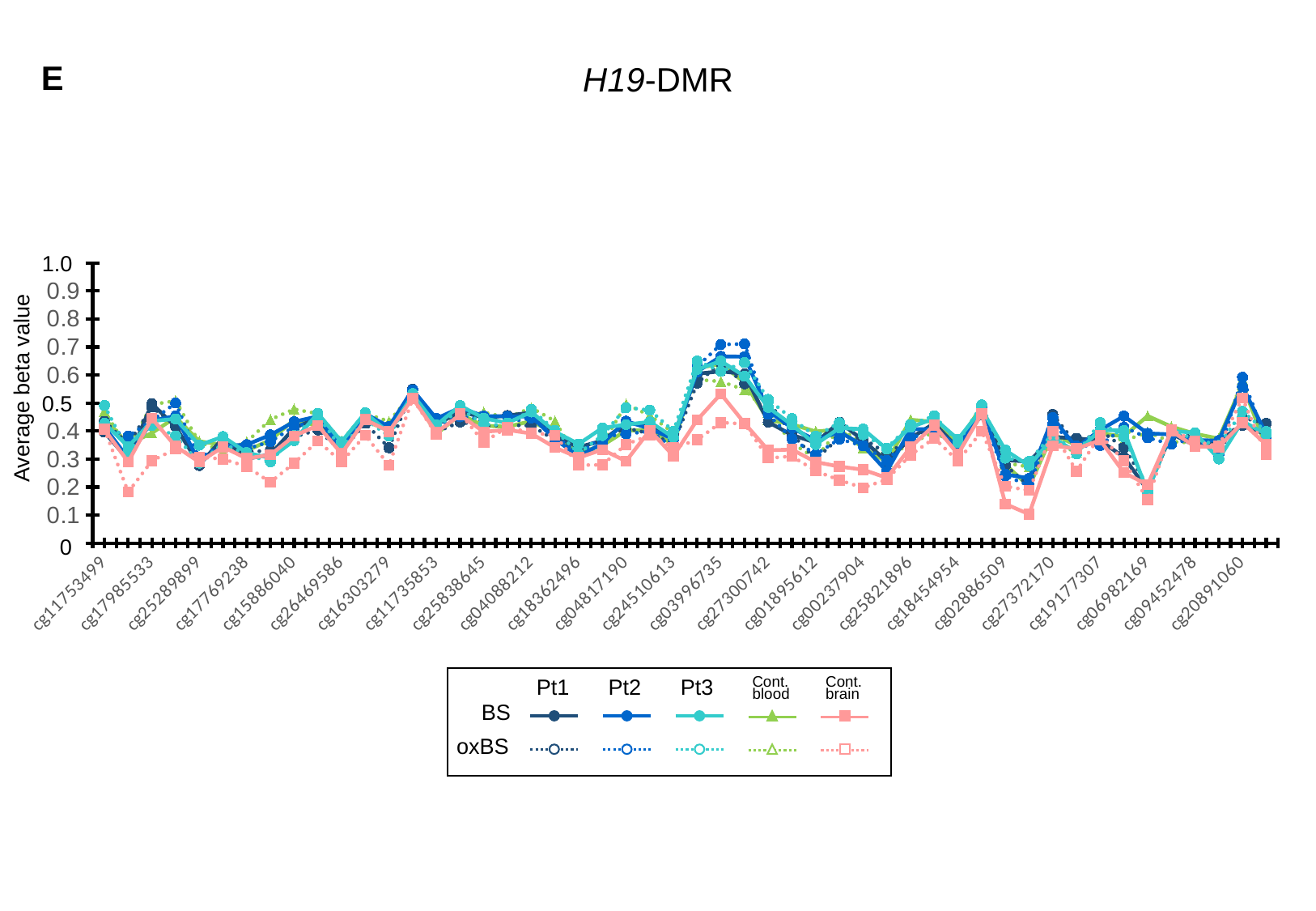

E
H19-DMR
### Chart
| Category | BS_pool | OX_pool | BS_869 | OX_869 | BS_3410 | OX_3410 | BS_5152 | OX_5152 | BS_Brain | OX_Brain |
|---|---|---|---|---|---|---|---|---|---|---|
| cg11753499 | 0.4441928 | 0.4708635 | 0.396041 | 0.4347154 | 0.41430715 | 0.41076605 | 0.4277646 | 0.4911435 | 0.4056785 | 0.40625435 |
| cg13210239 | 0.3524332 | 0.33418085 | 0.29691615 | 0.3534162 | 0.3027592 | 0.3817179 | 0.3439418 | 0.3253827 | 0.28926475 | 0.1817816 |
| cg17985533 | 0.39280465 | 0.4947704 | 0.4976166 | 0.4854339 | 0.43684805 | 0.436895 | 0.43571675 | 0.41939135 | 0.44528945 | 0.29338865 |
| cg15922305 | 0.4487543 | 0.50762335 | 0.4154026 | 0.4194143 | 0.45362895 | 0.49977085 | 0.44313935 | 0.38239445 | 0.34509595 | 0.3359197 |
| cg25289899 | 0.36320965 | 0.3583096 | 0.27899705 | 0.27678625 | 0.28902905 | 0.30120485 | 0.34933005 | 0.2855401 | 0.28905615 | 0.30664095 |
| cg25437674 | 0.35008205 | 0.32850485 | 0.3745987 | 0.36548645 | 0.3463685 | 0.3555716 | 0.37990195 | 0.35538925 | 0.342828 | 0.29979135 |
| cg17769238 | 0.32996105 | 0.3607671 | 0.3007958 | 0.3245288 | 0.3510812 | 0.3363349 | 0.3226767 | 0.3152795 | 0.30168415 | 0.2728031 |
| cg04975775 | 0.3674173 | 0.44057645 | 0.3158752 | 0.3306837 | 0.385902 | 0.3616853 | 0.3032341 | 0.2899686 | 0.31478475 | 0.21683445 |
| cg15886040 | 0.44064135 | 0.47811505 | 0.4104697 | 0.3787548 | 0.433116 | 0.41576385 | 0.3656726 | 0.38410265 | 0.3821927 | 0.28564225 |
| cg10154633 | 0.41755825 | 0.46164325 | 0.4471022 | 0.4037841 | 0.45238795 | 0.43628245 | 0.4631872 | 0.45521695 | 0.41997405 | 0.36414815 |
| cg26469586 | 0.3368546 | 0.3644854 | 0.33619565 | 0.32791005 | 0.3374905 | 0.3365356 | 0.36156135 | 0.3365683 | 0.31915055 | 0.2897278 |
| cg24409677 | 0.43722535 | 0.4636111 | 0.4482004 | 0.4274478 | 0.4617564 | 0.45725525 | 0.4651273 | 0.44178095 | 0.44058515 | 0.38467065 |
| cg16303279 | 0.40060845 | 0.43392475 | 0.41021275 | 0.340134 | 0.41566955 | 0.40649635 | 0.39328525 | 0.38167925 | 0.3982552 | 0.2778397 |
| cg02694715 | 0.5355977 | 0.53599695 | 0.5482415 | 0.5490877 | 0.54720425 | 0.54692885 | 0.5299511 | 0.5335694 | 0.5169391 | 0.5147695 |
| cg11735853 | 0.38856545 | 0.42272035 | 0.42232935 | 0.39583375 | 0.44436595 | 0.4209316 | 0.42114915 | 0.41184075 | 0.3951084 | 0.38772665 |
| cg01977486 | 0.45974055 | 0.45429865 | 0.47013385 | 0.4322808 | 0.4831091 | 0.4505598 | 0.4906663 | 0.48004885 | 0.45705345 | 0.46272605 |
| cg25838645 | 0.41884685 | 0.4665182 | 0.4473333 | 0.41760715 | 0.4530176 | 0.44270855 | 0.44558115 | 0.42417 | 0.3970089 | 0.35966785 |
| cg13581483 | 0.41532345 | 0.44758445 | 0.45505235 | 0.42149985 | 0.4521753 | 0.4313096 | 0.42889245 | 0.41995775 | 0.40380765 | 0.4143177 |
| cg04088212 | 0.43537925 | 0.48301955 | 0.46570595 | 0.4190053 | 0.4522545 | 0.44624075 | 0.46797115 | 0.47737955 | 0.390953 | 0.39280395 |
| cg01539474 | 0.3922062 | 0.43556025 | 0.38025555 | 0.3679699 | 0.37118725 | 0.3834876 | 0.3974936 | 0.402839 | 0.34074115 | 0.3852483 |
| cg18362496 | 0.3217563 | 0.2960935 | 0.34225545 | 0.32440995 | 0.30959645 | 0.3182669 | 0.3533659 | 0.3204484 | 0.3046684 | 0.27811865 |
| cg24605090 | 0.34817835 | 0.3525665 | 0.36514855 | 0.37567245 | 0.35551185 | 0.37850305 | 0.4109922 | 0.38378755 | 0.33324465 | 0.27898015 |
| cg04817190 | 0.4015627 | 0.4953901 | 0.42942215 | 0.41041345 | 0.4350628 | 0.38963805 | 0.4229872 | 0.4834732 | 0.29221975 | 0.35191945 |
| cg06749854 | 0.4033542 | 0.4560626 | 0.41860985 | 0.3906962 | 0.40807465 | 0.3986583 | 0.4326462 | 0.4747959 | 0.40235465 | 0.38554255 |
| cg24510613 | 0.35190705 | 0.39332695 | 0.3819335 | 0.3537062 | 0.37091715 | 0.3418394 | 0.37751595 | 0.39939565 | 0.3108597 | 0.3391455 |
| cg16675558 | 0.62386565 | 0.58339 | 0.60437505 | 0.5710392 | 0.61167595 | 0.6303099 | 0.61976725 | 0.64971175 | 0.4393875 | 0.36823415 |
| cg03996735 | 0.6397391 | 0.5767245 | 0.61308575 | 0.6434316 | 0.66620635 | 0.70872325 | 0.64953995 | 0.6133118 | 0.53268085 | 0.43030565 |
| cg18104242 | 0.5732053 | 0.54713595 | 0.60347985 | 0.56838735 | 0.66535275 | 0.7108187 | 0.59568185 | 0.6444343 | 0.42761635 | 0.4250327 |
| cg27300742 | 0.43915665 | 0.4670027 | 0.43150425 | 0.50624125 | 0.47171875 | 0.45953165 | 0.4842421 | 0.5128483 | 0.33105905 | 0.30320425 |
| cg25281616 | 0.4238852 | 0.35688195 | 0.385576 | 0.3886879 | 0.41083605 | 0.3718692 | 0.42159645 | 0.44433025 | 0.3335921 | 0.3096536 |
| cg01895612 | 0.3980941 | 0.30248345 | 0.35848 | 0.2992828 | 0.36690185 | 0.31644 | 0.35177655 | 0.38143035 | 0.28926925 | 0.25725585 |
| cg23476401 | 0.40521455 | 0.408264 | 0.4312719 | 0.37643155 | 0.39666435 | 0.37132145 | 0.4107108 | 0.42943195 | 0.2729868 | 0.2246509 |
| cg00237904 | 0.3393807 | 0.3580765 | 0.36737435 | 0.37423085 | 0.3482295 | 0.3466959 | 0.40652655 | 0.3866089 | 0.26172845 | 0.1968191 |
| cg06765785 | 0.28972435 | 0.30804005 | 0.29114165 | 0.3184738 | 0.2559933 | 0.2950187 | 0.33566645 | 0.33901275 | 0.23268185 | 0.22759255 |
| cg25821896 | 0.4403871 | 0.35359295 | 0.37433275 | 0.37093675 | 0.4066876 | 0.3759944 | 0.4135137 | 0.4227269 | 0.3423161 | 0.31420105 |
| cg25574978 | 0.43247385 | 0.3969699 | 0.42722715 | 0.41416865 | 0.40642925 | 0.3989471 | 0.444685 | 0.45468125 | 0.4206628 | 0.37446055 |
| cg18454954 | 0.3589856 | 0.34475815 | 0.33813105 | 0.33473785 | 0.3439081 | 0.34584815 | 0.37045435 | 0.35867135 | 0.31726315 | 0.2926896 |
| cg25579157 | 0.4874707 | 0.4710255 | 0.45827065 | 0.4731262 | 0.48107255 | 0.46395545 | 0.4772836 | 0.49289765 | 0.46186815 | 0.3988243 |
| cg02886509 | 0.28018495 | 0.2863104 | 0.29643 | 0.27687335 | 0.2453281 | 0.2391529 | 0.33133315 | 0.30305945 | 0.1394326 | 0.2026668 |
| cg02657360 | 0.2016913 | 0.27151445 | 0.29031 | 0.2041075 | 0.23088305 | 0.2055571 | 0.27557475 | 0.2916668 | 0.102914645 | 0.1874401 |
| cg27372170 | 0.38851615 | 0.36093 | 0.37842465 | 0.4587893 | 0.4478109 | 0.4237766 | 0.3756862 | 0.39040885 | 0.34718775 | 0.39841905 |
| cg22259242 | 0.3488313 | 0.3235278 | 0.3734487 | 0.3524188 | 0.34018695 | 0.34605225 | 0.3246201 | 0.3179808 | 0.33676885 | 0.25659035 |
| cg19177307 | 0.3905295 | 0.40246 | 0.36575505 | 0.4018979 | 0.4036334 | 0.3485516 | 0.40322895 | 0.43005315 | 0.3668475 | 0.3844271 |
| cg01585333 | 0.3812152 | 0.3651948 | 0.30647365 | 0.3401487 | 0.4531743 | 0.41339775 | 0.4015541 | 0.37922645 | 0.25112145 | 0.2936694 |
| cg06982169 | 0.45380415 | 0.3950307 | 0.18857965 | 0.20530415 | 0.39123815 | 0.37583495 | 0.1828894 | 0.1939669 | 0.20805045 | 0.1547254 |
| cg16574793 | 0.4156635 | 0.3617001 | 0.38968725 | 0.38311775 | 0.3876242 | 0.3527015 | 0.40047075 | 0.39877095 | 0.3996211 | 0.40479275 |
| cg09452478 | 0.3897521 | 0.357377 | 0.348537 | 0.3834829 | 0.3669786 | 0.3749677 | 0.39334915 | 0.3721922 | 0.34443305 | 0.3621867 |
| cg02864690 | 0.37294135 | 0.3374245 | 0.36567795 | 0.3310361 | 0.3658327 | 0.3170835 | 0.30016035 | 0.34834615 | 0.3427538 | 0.3451884 |
| cg20891060 | 0.5674893 | 0.58530775 | 0.42020145 | 0.4468751 | 0.55904805 | 0.5918394 | 0.43506405 | 0.47014 | 0.4296206 | 0.5179628 |
| cg19024989 | 0.34271775 | 0.3435703 | 0.38842235 | 0.42743515 | 0.3890858 | 0.3682294 | 0.3880031 | 0.39867935 | 0.3514264 | 0.31649615 |1.0
Average beta value
0.5
0
Pt1
Pt2
Pt3
Cont.
blood
BS
oxBS
Cont.
brain

## Slide 6
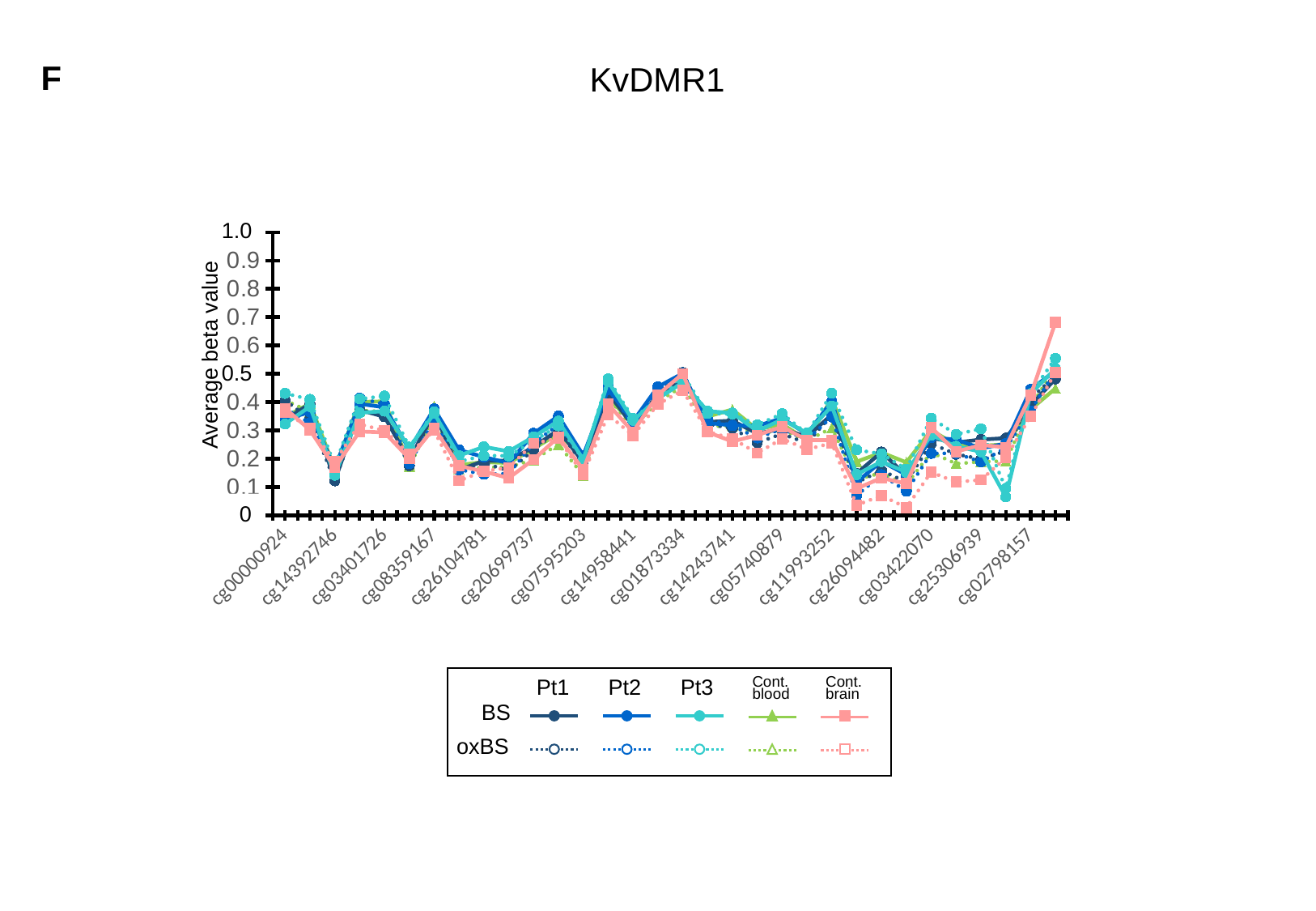

F
KvDMR1
### Chart
| Category | BS_pool | OX_pool | BS_869 | OX_869 | BS_3410 | OX_3410 | BS_5152 | OX_5152 | BS_Brain | OX_Brain |
|---|---|---|---|---|---|---|---|---|---|---|
| cg00000924 | 0.34734895 | 0.4054262 | 0.3377129 | 0.40594875 | 0.33913145 | 0.375704 | 0.3224293 | 0.430659 | 0.37750155 | 0.3634466 |
| cg11297256 | 0.3948888 | 0.3674031 | 0.39382095 | 0.3407413 | 0.36461635 | 0.3266352 | 0.3842771 | 0.40937365 | 0.3014089 | 0.30861915 |
| cg14392746 | 0.16386365 | 0.1528625 | 0.12219675 | 0.12192805 | 0.166315 | 0.1693665 | 0.1416804 | 0.1751413 | 0.1686479 | 0.189864675 |
| cg12077660 | 0.4005732 | 0.3705303 | 0.37229915 | 0.36367655 | 0.39364315 | 0.4140765 | 0.36199155 | 0.41144885 | 0.29592795 | 0.32181485 |
| cg03401726 | 0.40244285 | 0.36601545 | 0.34779395 | 0.35864245 | 0.38170755 | 0.39202395 | 0.3678191 | 0.42118275 | 0.2927017 | 0.299277 |
| cg16739686 | 0.1780629 | 0.17296925 | 0.19364345 | 0.1751126 | 0.2326421 | 0.1824725 | 0.23892725 | 0.2407385 | 0.20051265 | 0.21684855 |
| cg08359167 | 0.3857066 | 0.34798 | 0.36526645 | 0.3695401 | 0.37570755 | 0.3356211 | 0.3592799 | 0.3661829 | 0.30883445 | 0.2999698 |
| cg08446215 | 0.1776036 | 0.16311715 | 0.1601704 | 0.16165175 | 0.23109045 | 0.1612752 | 0.21073995 | 0.18961785 | 0.17474135 | 0.1221244 |
| cg26104781 | 0.18828075 | 0.16951475 | 0.19214215 | 0.18549555 | 0.20578265 | 0.1451626 | 0.2420534 | 0.2107383 | 0.1548532 | 0.1556911 |
| cg02219360 | 0.18479795 | 0.1706057 | 0.19327915 | 0.1566699 | 0.1840336 | 0.14700775 | 0.22588 | 0.21001735 | 0.1315177 | 0.16896585 |
| cg20699737 | 0.23619745 | 0.1963079 | 0.24098655 | 0.2316144 | 0.28966815 | 0.25499095 | 0.2771962 | 0.2622315 | 0.1972588 | 0.2542445 |
| cg26547719 | 0.2872001 | 0.2504021 | 0.3018261 | 0.30100445 | 0.3504455 | 0.3199033 | 0.3324987 | 0.31629185 | 0.2763707 | 0.27175905 |
| cg07595203 | 0.20590375 | 0.1399563 | 0.1869408 | 0.17866805 | 0.21135005 | 0.20081855 | 0.1968794 | 0.19691675 | 0.16118145 | 0.14448445 |
| cg09518720 | 0.4159012 | 0.4161333 | 0.43327545 | 0.4625302 | 0.4445783 | 0.4616931 | 0.47201565 | 0.481992 | 0.3928374 | 0.3551864 |
| cg14958441 | 0.324227 | 0.30828495 | 0.3152241 | 0.30909765 | 0.33183915 | 0.31256535 | 0.3352103 | 0.3425086 | 0.2940237 | 0.28164225 |
| cg27323091 | 0.41594745 | 0.4013619 | 0.4239006 | 0.4305834 | 0.45374645 | 0.4437313 | 0.4118825 | 0.40754635 | 0.4254479 | 0.39224095 |
| cg01873334 | 0.4740722 | 0.4555368 | 0.4853197 | 0.5042948 | 0.50119975 | 0.46375785 | 0.469138 | 0.4982188 | 0.50001315 | 0.4413222 |
| cg05816130 | 0.3486013 | 0.3126601 | 0.3296107 | 0.32205895 | 0.32599145 | 0.339867 | 0.3677022 | 0.3594514 | 0.2937254 | 0.2954849 |
| cg14243741 | 0.37371555 | 0.3178545 | 0.3343027 | 0.30705895 | 0.3178159 | 0.3163918 | 0.36132615 | 0.35794365 | 0.26074545 | 0.27052225 |
| cg21137515 | 0.30310895 | 0.2844971 | 0.28878795 | 0.25626525 | 0.31584475 | 0.2734276 | 0.2974969 | 0.3193317 | 0.28320995 | 0.21962865 |
| cg05740879 | 0.3221395 | 0.3028211 | 0.30505465 | 0.2879936 | 0.34264335 | 0.3056124 | 0.3382111 | 0.3586882 | 0.31266725 | 0.27043675 |
| cg01893176 | 0.26522115 | 0.2635784 | 0.27728125 | 0.2541832 | 0.2866787 | 0.26682445 | 0.29051935 | 0.28813185 | 0.2656836 | 0.2328598 |
| cg11993252 | 0.4122852 | 0.30830345 | 0.35566625 | 0.3555588 | 0.4021822 | 0.3476063 | 0.38601075 | 0.43139555 | 0.2656472 | 0.2549587 |
| cg11666921 | 0.18954865 | 0.12673685 | 0.14769005 | 0.115712465 | 0.1171952 | 0.070226435 | 0.14419205 | 0.23096315 | 0.094783155 | 0.036098105 |
| cg26094482 | 0.22165535 | 0.1500429 | 0.2234174 | 0.158359 | 0.19076305 | 0.1434142 | 0.18982115 | 0.2168112 | 0.13178195 | 0.06972682 |
| cg26908876 | 0.18689075 | 0.108907925 | 0.1398322 | 0.11758997 | 0.14327245 | 0.08518956 | 0.15234305 | 0.1633156 | 0.112049685 | 0.02751387 |
| cg03422070 | 0.3005546 | 0.22204355 | 0.2731436 | 0.2532081 | 0.27403875 | 0.21883115 | 0.2835662 | 0.34348875 | 0.3098282 | 0.1523894 |
| cg15651941 | 0.24893195 | 0.18240105 | 0.25693625 | 0.21578175 | 0.26758615 | 0.21873805 | 0.2436499 | 0.2859903 | 0.2247831 | 0.11769205 |
| cg25306939 | 0.2455783 | 0.18922495 | 0.2675284 | 0.19906555 | 0.2363868 | 0.1885622 | 0.2245927 | 0.30544395 | 0.24826155 | 0.125050845 |
| cg06288089 | 0.2506021 | 0.19160315 | 0.27254135 | 0.2268697 | 0.25327225 | 0.2385046 | 0.06522071 | 0.09487606 | 0.2403461 | 0.2027876 |
| cg02798157 | 0.37404095 | 0.38369945 | 0.388667 | 0.4125083 | 0.4444812 | 0.3684675 | 0.42969235 | 0.42829435 | 0.4238628 | 0.34983415 |
| cg27604721 | 0.4480971 | 0.5224641 | 0.48179815 | 0.4924601 | 0.5103602 | 0.51193455 | 0.51779295 | 0.5547064 | 0.68117385 | 0.5046494 |1.0
Average beta value
0.5
0
Pt1
Pt2
Pt3
Cont.
blood
BS
oxBS
Cont.
brain

## Slide 7
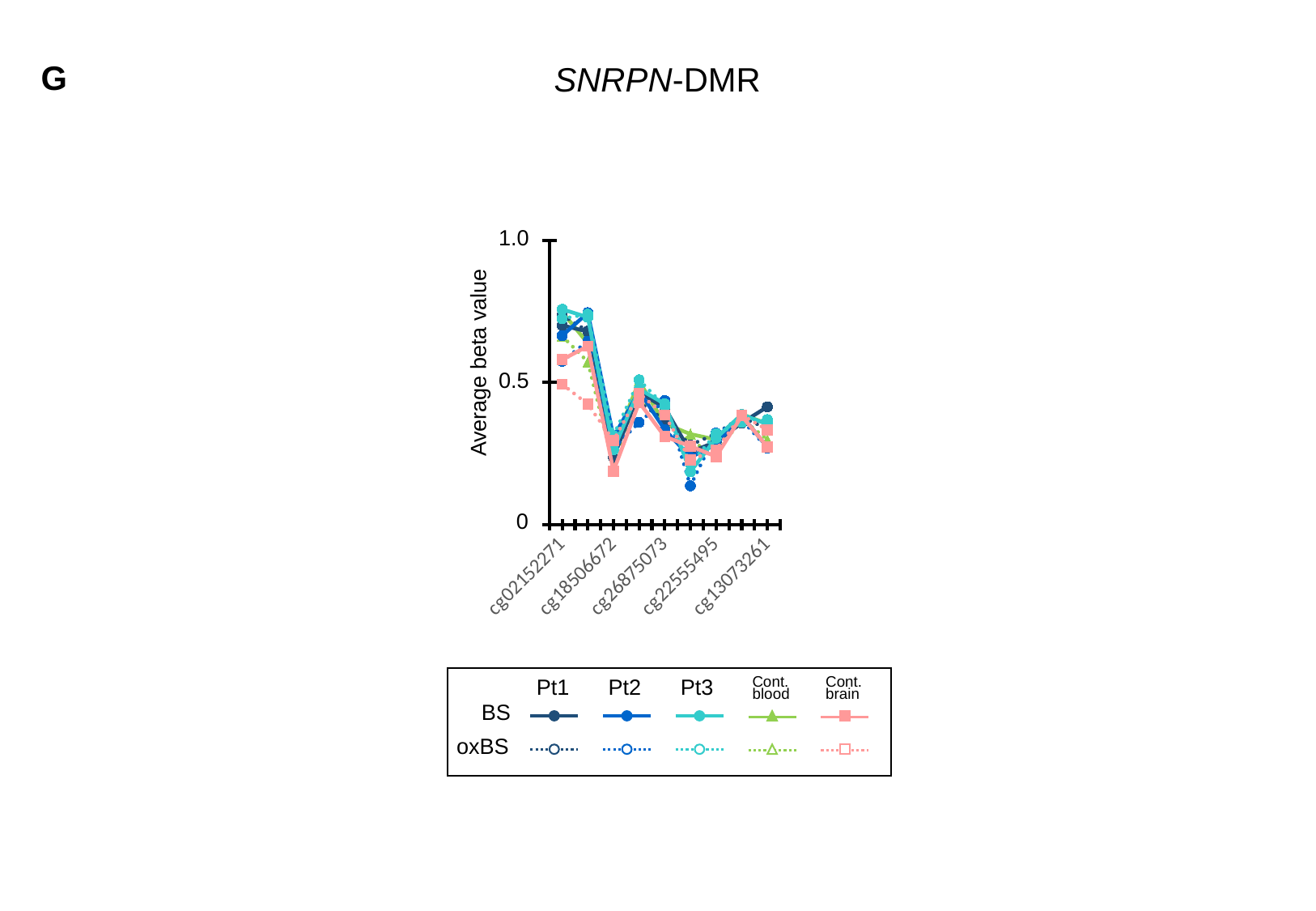

G
SNRPN-DMR
### Chart
| Category | BS_pool | OX_pool | BS_869 | OX_869 | BS_3410 | OX_3410 | BS_5152 | OX_5152 | BS_Brain | OX_Brain |
|---|---|---|---|---|---|---|---|---|---|---|
| cg02152271 | 0.7474632 | 0.66323965 | 0.70062665 | 0.7367497 | 0.6642653 | 0.5743617 | 0.757061 | 0.7243867 | 0.58011075 | 0.49415705 |
| cg12298755 | 0.63778965 | 0.57241855 | 0.6788875 | 0.6801507 | 0.7449858 | 0.6452054 | 0.72939635 | 0.73974085 | 0.62762585 | 0.42349865 |
| cg18506672 | 0.2762966 | 0.20782175 | 0.23673855 | 0.29317365 | 0.30738005 | 0.26866275 | 0.2636538 | 0.31278465 | 0.1884102 | 0.29584225 |
| cg02125271 | 0.50453415 | 0.448889 | 0.464646 | 0.42946705 | 0.46978575 | 0.35983125 | 0.47676 | 0.50895235 | 0.4303011 | 0.46157535 |
| cg26875073 | 0.3545769 | 0.35448515 | 0.4115236 | 0.3680798 | 0.3340467 | 0.4364625 | 0.42482995 | 0.4167138 | 0.30940795 | 0.38508365 |
| cg22159025 | 0.31857375 | 0.2926809 | 0.25542935 | 0.280412 | 0.2369118 | 0.136509255 | 0.18606905 | 0.21397765 | 0.27610175 | 0.22705415 |
| cg22555495 | 0.29899775 | 0.2493137 | 0.29127075 | 0.3200068 | 0.28265405 | 0.3219367 | 0.3042037 | 0.3198344 | 0.23951045 | 0.2633036 |
| cg01614564 | 0.38863325 | 0.3723494 | 0.3577338 | 0.3790427 | 0.3835892 | 0.3685593 | 0.3873659 | 0.359282 | 0.3822223 | 0.38525445 |
| cg13073261 | 0.3548349 | 0.2989769 | 0.41444415 | 0.336108 | 0.2720697 | 0.26934765 | 0.3530656 | 0.36942375 | 0.273696 | 0.33334355 |1.0
Average beta value
0.5
0
Pt1
Pt2
Pt3
Cont.
blood
BS
oxBS
Cont.
brain

## Slide 8
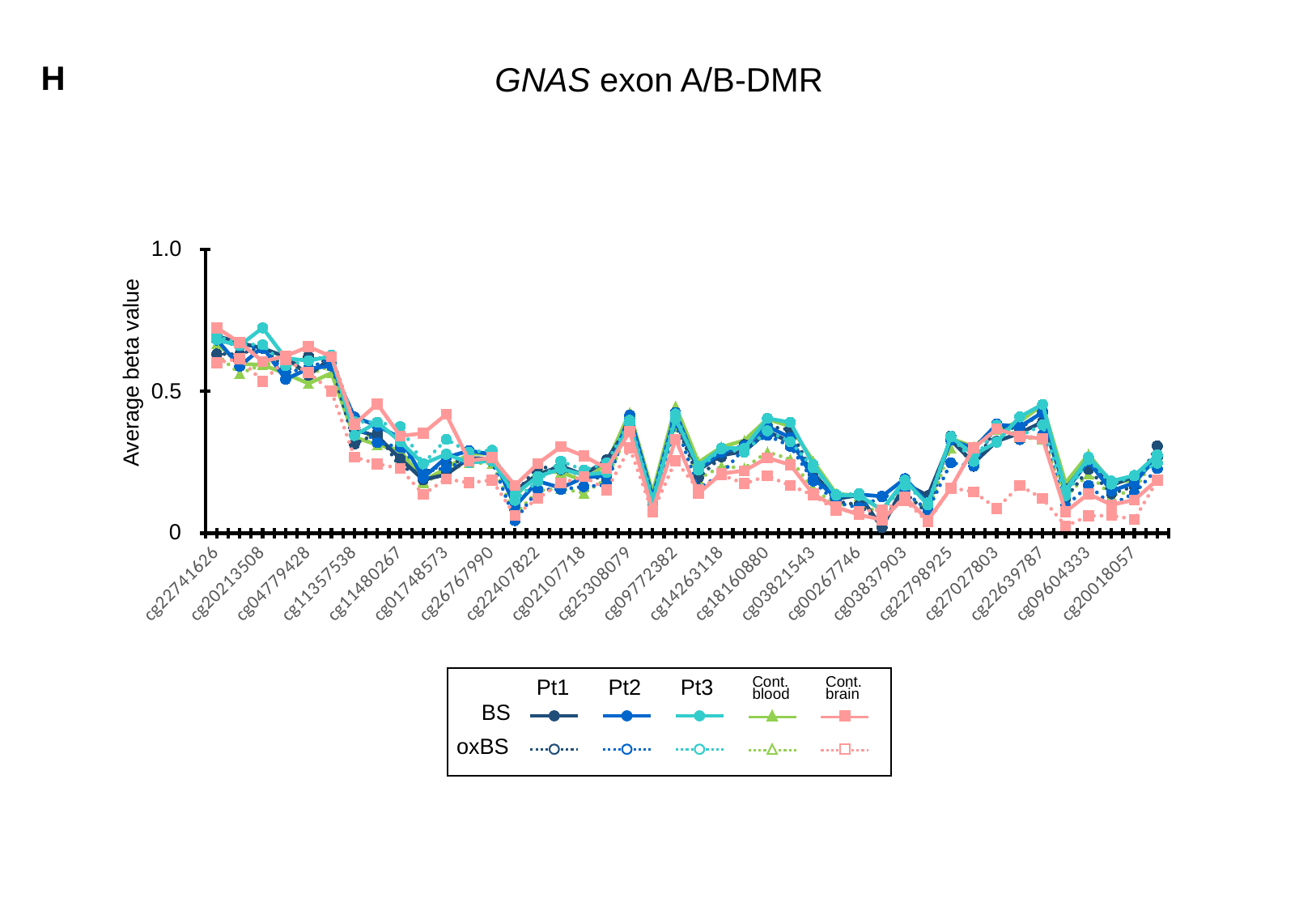

H
GNAS exon A/B-DMR
### Chart
| Category | BS_pool | OX_pool | BS_869 | OX_869 | BS_3410 | OX_3410 | BS_5152 | OX_5152 | BS_Brain | OX_Brain |
|---|---|---|---|---|---|---|---|---|---|---|
| cg22741626 | 0.66935765 | 0.62886475 | 0.6956882 | 0.6315896 | 0.68250995 | 0.68100895 | 0.6816542 | 0.69528685 | 0.72358475 | 0.6008402 |
| cg18997188 | 0.5965856 | 0.562727 | 0.6704036 | 0.63050985 | 0.58816805 | 0.65981405 | 0.66129905 | 0.66619095 | 0.67217215 | 0.61507335 |
| cg20213508 | 0.59361225 | 0.59465265 | 0.65201215 | 0.65917095 | 0.65657445 | 0.6525322 | 0.7238729 | 0.664359 | 0.6054223 | 0.534837 |
| cg26791489 | 0.56254035 | 0.6027856 | 0.6212972 | 0.5796841 | 0.5423674 | 0.56703835 | 0.61658095 | 0.5905547 | 0.6242592 | 0.61042155 |
| cg04779428 | 0.52705675 | 0.55807645 | 0.5568667 | 0.623247 | 0.5800849 | 0.58156775 | 0.60804985 | 0.6077006 | 0.65738635 | 0.56589545 |
| cg04019914 | 0.56460725 | 0.59128445 | 0.61732135 | 0.5975834 | 0.5896358 | 0.61646255 | 0.62383845 | 0.62736135 | 0.62234975 | 0.50004055 |
| cg11357538 | 0.3387063 | 0.3668603 | 0.36517395 | 0.31369805 | 0.40945665 | 0.383386 | 0.3440766 | 0.3937213 | 0.3838903 | 0.26815305 |
| cg20008140 | 0.31084365 | 0.33158915 | 0.34084185 | 0.3523018 | 0.3781298 | 0.31915675 | 0.39108915 | 0.39065265 | 0.4557482 | 0.24421575 |
| cg11480267 | 0.29208725 | 0.2808056 | 0.26244555 | 0.2319633 | 0.33578385 | 0.30280865 | 0.3221281 | 0.37537365 | 0.3422619 | 0.2274686 |
| cg10011623 | 0.1834801 | 0.176751 | 0.1880064 | 0.2110519 | 0.19527875 | 0.21548935 | 0.2442257 | 0.24511915 | 0.35214415 | 0.13675185 |
| cg01748573 | 0.22884485 | 0.2174359 | 0.2064906 | 0.24588965 | 0.2665863 | 0.23055495 | 0.2782246 | 0.3307141 | 0.4188016 | 0.19118415 |
| cg17334845 | 0.2791563 | 0.2484908 | 0.2640236 | 0.2597082 | 0.29070265 | 0.25851435 | 0.24864205 | 0.28550775 | 0.25518295 | 0.17736635 |
| cg26767990 | 0.2809678 | 0.24503895 | 0.26261945 | 0.26654165 | 0.27753955 | 0.25341445 | 0.25442105 | 0.2919761 | 0.2659216 | 0.18696585 |
| cg17652507 | 0.1270871 | 0.065346735 | 0.14994005 | 0.1393314 | 0.09440804 | 0.044463595 | 0.1374202 | 0.11560772 | 0.16792318 | 0.062157925 |
| cg22407822 | 0.2162917 | 0.14995595 | 0.2063202 | 0.2349214 | 0.1830992 | 0.1537156 | 0.19978845 | 0.18398995 | 0.2455619 | 0.123034425 |
| cg07341934 | 0.2150665 | 0.15745625 | 0.23658625 | 0.22414405 | 0.159450445 | 0.1539711 | 0.22610215 | 0.2536639 | 0.3045279 | 0.1765891 |
| cg02107718 | 0.18380785 | 0.14131735 | 0.20531565 | 0.2042448 | 0.19570645 | 0.16397805 | 0.20628785 | 0.2224621 | 0.2720377 | 0.1995589 |
| cg23496597 | 0.24536015 | 0.19120015 | 0.25826125 | 0.18871575 | 0.2027842 | 0.17005145 | 0.2133349 | 0.24695525 | 0.2280059 | 0.1518685 |
| cg25308079 | 0.425619 | 0.36942075 | 0.3839909 | 0.38877795 | 0.4160066 | 0.4001869 | 0.38654475 | 0.3979916 | 0.3579089 | 0.3008599 |
| cg03014008 | 0.14294255 | 0.089100845 | 0.118199385 | 0.0983248 | 0.1261907 | 0.09802944 | 0.111079555 | 0.124223875 | 0.07929321 | 0.073249775 |
| cg09772382 | 0.4480058 | 0.3742144 | 0.3971373 | 0.3929151 | 0.42526905 | 0.38737875 | 0.4025998 | 0.4203196 | 0.331316 | 0.2558189 |
| cg15222215 | 0.24939595 | 0.19174355 | 0.22292455 | 0.19532675 | 0.226045 | 0.1560863 | 0.2358505 | 0.2229412 | 0.1401104 | 0.153324 |
| cg14263118 | 0.3032197 | 0.23514095 | 0.27431465 | 0.2693483 | 0.284167 | 0.2083168 | 0.30011555 | 0.2972343 | 0.2100195 | 0.2050542 |
| cg11244758 | 0.32690455 | 0.2292998 | 0.28846055 | 0.31186885 | 0.30814055 | 0.29937855 | 0.2988905 | 0.2853973 | 0.2200218 | 0.17462725 |
| cg18160880 | 0.4018737 | 0.28589165 | 0.35811535 | 0.37689375 | 0.37618895 | 0.34484835 | 0.4043946 | 0.3606524 | 0.26544775 | 0.2020459 |
| cg05926269 | 0.3766476 | 0.26068875 | 0.3211288 | 0.36420205 | 0.3369006 | 0.3066026 | 0.39048065 | 0.32233315 | 0.24091025 | 0.1678482 |
| cg03821543 | 0.25400735 | 0.1572841 | 0.21179925 | 0.2085708 | 0.19100975 | 0.18388705 | 0.24164905 | 0.23107675 | 0.1376626 | 0.13413835 |
| cg01538522 | 0.13892815 | 0.098368955 | 0.12029095 | 0.112077175 | 0.12845995 | 0.107353135 | 0.13433393 | 0.135812 | 0.09187119 | 0.08003209 |
| cg00267746 | 0.1314355 | 0.105327605 | 0.1344501 | 0.09934571 | 0.13642775 | 0.08968672 | 0.132646245 | 0.13922225 | 0.06483409 | 0.076456975 |
| cg09885502 | 0.081798815 | 0.06722009 | 0.01949848 | 0.033788545 | 0.12971104 | 0.12783014 | 0.08185904 | 0.081721495 | 0.046807275 | 0.08233027 |
| cg03837903 | 0.1838831 | 0.1348898 | 0.1749306 | 0.14143295 | 0.19200675 | 0.14466645 | 0.18688875 | 0.16847005 | 0.1269522 | 0.11502463 |
| cg23159236 | 0.11276905 | 0.080096305 | 0.13183905 | 0.06992821 | 0.10610499 | 0.080530665 | 0.099754905 | 0.106856555 | 0.045719225 | 0.039054375 |
| cg22798925 | 0.33335975 | 0.30057485 | 0.3279281 | 0.3423615 | 0.3287527 | 0.24783825 | 0.3397789 | 0.34065375 | 0.1585782 | 0.15987135 |
| cg20126878 | 0.30498405 | 0.3033924 | 0.24755205 | 0.2357965 | 0.29850395 | 0.23615965 | 0.27602575 | 0.25284605 | 0.30068005 | 0.14537615 |
| cg27027803 | 0.3648321 | 0.3816886 | 0.3239994 | 0.3765101 | 0.3828097 | 0.38522845 | 0.32091005 | 0.3803125 | 0.3671968 | 0.08692119 |
| cg08997444 | 0.39264975 | 0.3364512 | 0.35227395 | 0.3674257 | 0.3749225 | 0.32998445 | 0.40982055 | 0.34217375 | 0.34133635 | 0.16766035 |
| cg22639787 | 0.45245045 | 0.33250785 | 0.3921015 | 0.42764905 | 0.42564185 | 0.3461874 | 0.453951 | 0.3813427 | 0.33317035 | 0.1217586 |
| cg05960039 | 0.17693005 | 0.1280736 | 0.1553901 | 0.1232785 | 0.15010225 | 0.094204785 | 0.1508205 | 0.1325026 | 0.07571932 | 0.025110385 |
| cg09604333 | 0.27765945 | 0.2087127 | 0.26022095 | 0.2251105 | 0.25311455 | 0.16810105 | 0.26763805 | 0.25989255 | 0.138367 | 0.060521735 |
| cg06047881 | 0.1772241 | 0.1327976 | 0.16900945 | 0.13927325 | 0.1489635 | 0.10055602 | 0.1836927 | 0.1730088 | 0.09965518 | 0.062022415 |
| cg20018057 | 0.1874663 | 0.14946125 | 0.19941325 | 0.165499 | 0.17715485 | 0.14058025 | 0.2032041 | 0.20163065 | 0.1172664 | 0.04840305 |
| cg10748817 | 0.2702783 | 0.28430605 | 0.26797535 | 0.3073101 | 0.2724475 | 0.22799725 | 0.27664145 | 0.24685855 | 0.18747445 | 0.18605895 |1.0
Average beta value
0.5
0
Pt1
Pt2
Pt3
Cont.
blood
BS
oxBS
Cont.
brain

## Slide 9
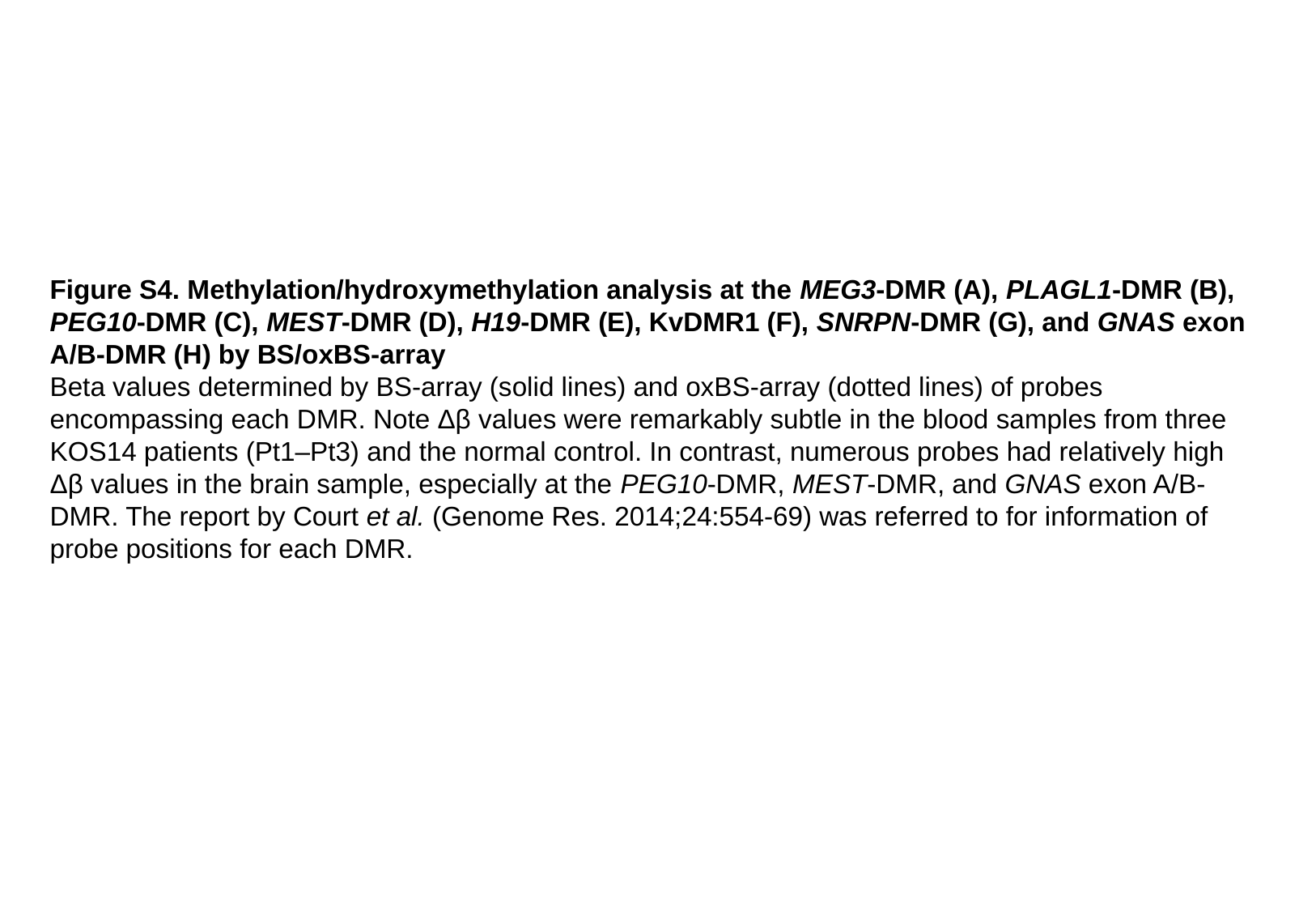

Figure S4. Methylation/hydroxymethylation analysis at the MEG3-DMR (A), PLAGL1-DMR (B), PEG10-DMR (C), MEST-DMR (D), H19-DMR (E), KvDMR1 (F), SNRPN-DMR (G), and GNAS exon A/B-DMR (H) by BS/oxBS-array
Beta values determined by BS-array (solid lines) and oxBS-array (dotted lines) of probes encompassing each DMR. Note Δβ values were remarkably subtle in the blood samples from three KOS14 patients (Pt1–Pt3) and the normal control. In contrast, numerous probes had relatively high Δβ values in the brain sample, especially at the PEG10-DMR, MEST-DMR, and GNAS exon A/B-DMR. The report by Court et al. (Genome Res. 2014;24:554-69) was referred to for information of probe positions for each DMR.
