# Supplementary material for: Exploration of hydroxymethylation in Kagami-Ogata syndrome caused by hypermethylation of imprinting control regions
Source: Clin Epigenetics. 2015 Aug 28;7(1):90. doi: 10.1186/s13148-015-0124-y (PMC4552283; doi:10.1186/s13148-015-0124-y)
Supplement: Additional file 7: Figure S5. — Gel electropherograms showing appropriate digestion pattern of spike-in controls. (PPB 3.31 mb) [file 13148_2015_124_MOESM7_ESM.pptx]

## Slide 1
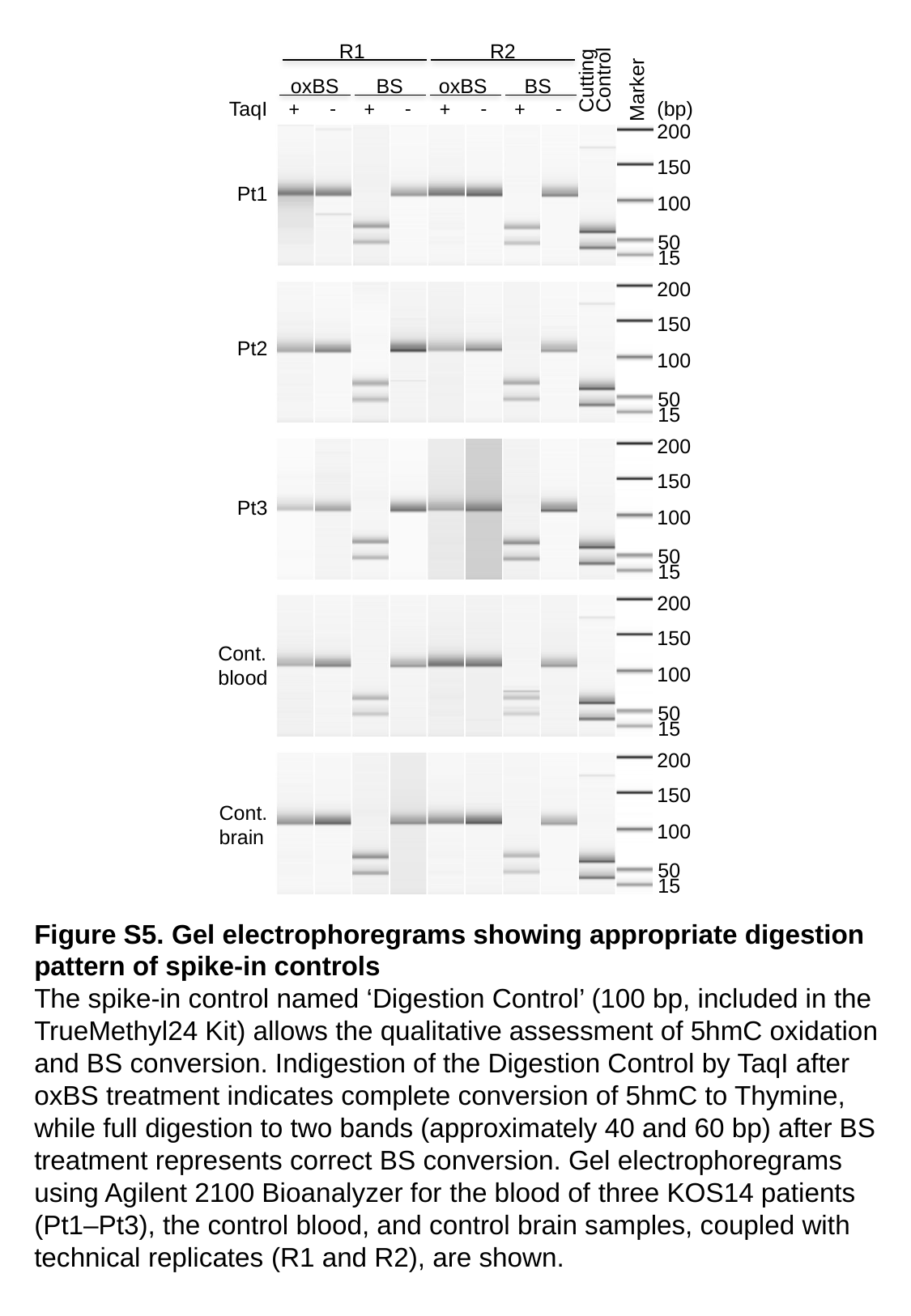

R1
R2
Cutting
Control
oxBS
BS
oxBS
BS
Marker
TaqI
+
-
+
-
+
-
+
-
(bp)
200
150
100
50
15
Pt1
200
150
100
50
15
Pt2
200
150
100
50
15
Pt3
200
150
100
50
15
Cont.
blood
200
150
100
50
15
Cont.
brain
Figure S5. Gel electrophoregrams showing appropriate digestion pattern of spike-in controls
The spike-in control named ‘Digestion Control’ (100 bp, included in the TrueMethyl24 Kit) allows the qualitative assessment of 5hmC oxidation and BS conversion. Indigestion of the Digestion Control by TaqI after oxBS treatment indicates complete conversion of 5hmC to Thymine, while full digestion to two bands (approximately 40 and 60 bp) after BS treatment represents correct BS conversion. Gel electrophoregrams using Agilent 2100 Bioanalyzer for the blood of three KOS14 patients (Pt1–Pt3), the control blood, and control brain samples, coupled with technical replicates (R1 and R2), are shown.
